# Exploring Biomarkers in Type 2 Diabetes Mellitus versus Normoglycemia Identified through High-Throughput Proteomics: A Systematic Review and Meta-Analysis

**DOI:** 10.1021/acs.jproteome.5c00773

**Published:** 2025-11-30

**Authors:** Julia García-Currás, Raquel Pérez-Lois, Guillermo L. Taboada, María P. Pata

**Affiliations:** † Universidade da Coruña (UDC), Grupo Arquitectura de Computadores, Centro de Investigación en Tecnologías de la Información y las Comunicaciones (CITIC), Elviña, A Coruña 15071, Spain; ‡ Biostatech, Advice, Training & Innovation in Biostatistics, Ames(A Coruña) 15895, Spain; § Grupo Fisiopatología Endocrina, Área de Endocrinología, Instituto de Investigación Sanitaria de Santiago de Compostela (IDIS), Complexo Hospitalario Universitario de Santiago (SERGAS), Travesía da Choupana s/n, Santiago de Compostela, Galicia 15706, Spain; ∥ CIBER Fisiopatología de la Obesidad y Nutrición (CIBERobn), Av Monforte de Lemos 3-5, Madrid 28029, Spain

**Keywords:** type 2 diabetes mellitus, proteomic biomarkers, high-throughput proteomics, omics-based meta-analysis, random-effects meta-analysis

## Abstract

Recent advances in proteomics have enabled the identification of early protein biomarkers and metabolic disturbances associated with type 2 diabetes (T2D), a major global health challenge. This systematic review and meta-analysis synthesize evidence from 27 studies comparing proteomic profiles of individuals with T2D and normoglycemic controls, selected from 2,422 initial records. The QUADOMICS assessment showed good methodological reporting for sample handling and proteomic analysis (>70% of studies), but over 60% lacked information on confounding clinical factors and biomarker validation. A qualitative synthesis focused on 85 recurrently reported proteins (≥8 studies), which showed strong interconnectivity and were involved in immune response, lipid–protein organization, detoxification, proteolysis, and coagulation, key pathways implicated in T2D. An omics-based meta-analysis identified seven promising protein biomarkers for T2D related to lipid/glucose metabolism (Q12907_LMAN2, P02652_POA2, P07602_PSPA, P09622_DLD); cell binding/adhesion (P12109_COL6A1, P12830_CDH1); and translational regulation and mitochondrial function (P35232_PHB). Random-effects meta-analysis revealed variation in effect sizes across studies for previously highlighted biomarkers, but three of them (P02763_ORM1, P00738_HP, P25311_AZGP1) exhibited considerable consistency. To enhance accessibility and further exploration of findings, we provide the interactive web tool ***metaMarkersT2D***: https://jgcurras.shinyapps.io/metaMarkersT2D/.

## Graphical abstract

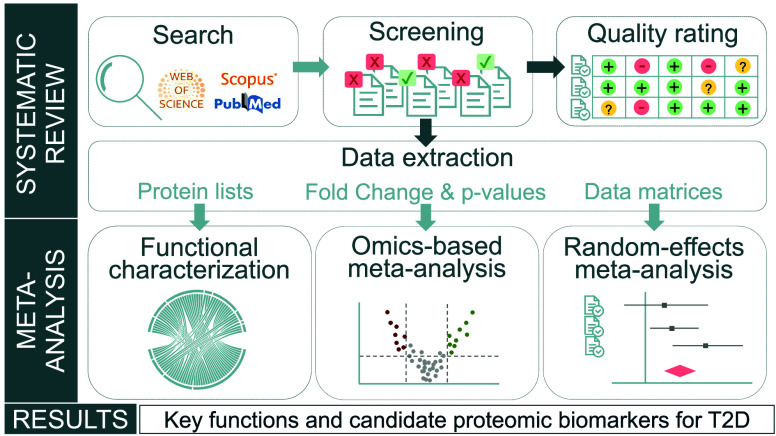

## Introduction

Type 2 diabetes (T2D) is a complex and progressive metabolic disorder characterized by persistent hyperglycemia, resulting from insulin resistance and progressive pancreatic β-cell dysfunction.^1^ Representing approximately 90% of all diabetes cases worldwide, T2D has reached epidemic proportions, mainly driven by population aging, sedentary lifestyles, and poor dietary habits.^2^ The latest report from the International Diabetes Federation indicates that over 589 million adults aged 20–79 are currently living with diabetes, a number projected to exceed 850 million by 2050.^2^ T2D is associated with a high risk of microvascular complications (retinopathy, nephropathy, neuropathy) and macrovascular conditions (coronary heart disease, stroke), significantly increasing premature mortality and healthcare costs.^3–8^ Despite advancements in therapeutics, early detection and prevention remain critical challenges, especially given that the metabolic alterations underlying the disease may begin years before a clinical diagnosis is established.^1,9^ Due to this prolonged asymptomatic phase, there is an urgent need for reliable biomarkers capable of identifying at-risk individuals before irreversible metabolic damage occurs.

Biomarkers are measurable indicators of biological processes, disease states, and therapeutic responses. Given T2D’s primarily environmental etiology and the significant improvement of proteomic technologies in recent years, proteins represent especially promising biomarker candidates. As functional executors of cellular processes, proteins may reflect early pathophysiological changes and serve as potential therapeutic targets. Protein biomarker research in T2D has been widely explored and generally involves a comprehensive proteome screening of target tissues, followed by differential abundance analysis (DAA) to identify dysregulated or disease-specific proteins associated with T2D. In this context, targeted techniques such as ELISA (Enzyme-Linked Immunosorbent Assay), Western blotting, immunoprecipitation assays, or SRM/MRM (Selected/Multiple Reaction Monitoring) MS (Mass Spectrometry) are valuable for validation, but their limited scope makes them unsuitable for discovery-phase studies.^10^ Traditional proteomic methods for biomarker discovery typically involved two-dimensional electrophoresis (2-DE) or liquid chromatography (LC) for protein separation, followed by identification using Matrix-Assisted Laser Desorption/Ionization (MALDI) MS or Surface-Enhanced Laser Desorption/Ionization (SELDI) MS.^11–16^ However, these early approaches had considerable limitations, as they only enabled the identification of a small subset of proteins associated with T2D. Although an additional quantification step was included using gel staining^17–20^ or differential fluorescence labeling (two-dimensional difference gel electrophoresis or 2D-DIGE)^21–24^ to identify differentially abundant proteins, detection remained restricted to a limited number of molecules. In recent years, more sensitive targeted proteomic platforms such as Olink, a proximity extension assay that uses qPCR for protein quantification,^25–28^ and SomaScan, which relies on modified DNA aptamers that selectively bind proteins whose abundance is quantified via DNA arrays, qPCR, or sequencing,^29–34^ have allowed for deeper proteomic exploration. Despite their broader coverage, these are still targeted proteomic approaches: target proteins are selected prior to analysis, provided that specific antibodies or aptamers are available. Shotgun proteomics, a high-throughput and untargeted approach, has greatly expanded protein identification capacity.^35^ Operating in data-dependent acquisition (DDA) mode, shotgun involves selecting the most abundant precursor ions in the MS1 scan for MS2 spectra recording,^35^ which limits reproducibility and the detection of low-abundance proteins. These challenges are addressed by label-free data-independent acquisition (DIA) methods, which perform a windowed full fragmentation of precursor ions in MS2,^36^ offering a promising alternative for T2D biomarker discovery. The DIA approach enhances reproducibility and consistency across samples by avoiding precursor ion selection bias and enabling comprehensive, untargeted proteome coverage. However, this method requires individual sample analysis, increasing costs, and being a relatively recent approach, few studies applying DIA for T2D biomarker discovery exist to date, with none published before 2015.^10^ Considering both methodological strengths and limitations, shotgun and DIA-based proteomics currently offer the most comprehensive and informative strategies for identifying protein biomarkers in T2D. Their broad proteome coverage and their enhanced ability to capture multiple proteins provide valuable insights into the disease’s complex pathogenesis.

Although launching another primary study might seem a logical next step, the growing number of individual reports risks contributing to fragmentation rather than synthesis. Without integration, the proliferation of candidate biomarkers can delay translation from discovery into clinical application, particularly in the form of validated multibiomarker panels.^37^ As proposed by Tans et al., 2018,^37^ such a panel should integrate diverse biomarkers across omics layers (transcriptomics, metabolomics, proteomics), reflecting key systemic changes in T2D and integrated via systems biology models. Achieving this ambitious aim requires first integrating biomarkers within each omics dimension to better understand primary alterations and highlight the most representative markers. In this context, proteomic reviews of T2D biomarkers are essential to consolidating knowledge and providing a comprehensive overview of individual studies. Although a relevant number of reviews on T2D proteomic biomarker discovery are available in the literature,^38–44^ many adopt a narrative format without applying strict inclusion criteria, which can make it more challenging to assess the strength of the evidence. In some cases, data from different organisms or sample types are discussed together,^41,42^ which may lead to misinterpretation if those distinctions are not explicitly stated. Additionally, key methodological details such as the characteristics of the control groups or the direction and magnitude of biomarker changes are not always reported, potentially limiting cross-study comparability.^39,40,43,44^ In contrast, quantitative meta-analyses offer a rigorous, transparent framework to identify the most consistent and statistically robust biomarkers. Yet, such analyses remain rare for proteomic T2D studies. A search in the PROSPERO database reveals few registered entries in this domain.^45^ One key barrier has been data availability: many proteomic studies report only summary statistics (e.g., fold change (FC), *p*-values from DAA) for a subset of detected proteins without making raw data accessible. Recently, Llambrich et al., 2022 ^46^ developed a novel aggregation method, implemented in the Amanida R package, which overcomes these challenges by leveraging available metrics and weighting them by study sample sizes to enable more robust meta-analytic integration, maximizing the use of available proteomic data.

In this study, we address the urgent need for evidence integration in T2D proteomics by conducting a systematic review and meta-analysis focused on differentially abundant proteins between individuals with T2D and normoglycemic controls. We include only nontargeted high-throughput proteomic studies employing comprehensive acquisition strategies, particularly shotgun and DIA-based approaches. Through a combination of a rigorous study selection process with both qualitative synthesis and quantitative aggregation, our goal is to identify robust and reproducible protein biomarkers, detect key altered pathways in T2D, and support the development of clinically relevant multibiomarker panels.

## Methods

### Study Design

A systematic review and meta-analysis were conducted to address the following research question incorporating all PICO (Population, Intervention, Comparison, Outcome) components: *Which proteins exhibit differential abundance between individuals with type 2 diabetes and normoglycemic controls, as identified through high-throughput proteomic analysis?* A detailed protocol was registered in PROSPERO^45^ (CRD42025648346) prior to the analysis, and the review was conducted in accordance with the PRISMA guidelines.

### Search Strategy

Two consecutive searches were conducted in the following databases: PubMed Central (PMC), Scopus, and Web of Science (WOS). The first search (February 12, 2025) was used to refine the initial search strategy, followed by a final search using a more precise equation (February 13–14, 2025). For the refinement strategy,^47^ results from the first search were used to identify additional relevant terms (from titles, abstracts, and keywords) (Supplementary Table S1). These terms were then manually grouped, selected, and incorporated into the final search equation (Supplementary Table S2).

### Study Selection

PubMed references downloaded in CSV format included only limited article information, lacking key data necessary for screening such as abstracts. While the PubMed format contains all information for each article, it is not structured as a table, making it difficult to work with. To convert the PubMed format into a tabular structure, a web application (pubmedToCsv) was developed,^48^ and this final table can be downloaded in CSV format.

Individual results for the three databases were merged, and duplicate studies were removed using the DOI as the primary identifier. Study selection was conducted with meticulous attention to the inclusion and exclusion criteria defined in Supplementary Table S3. All articles involving both a T2D group and a control group were considered in the selection process, even if the primary focus of the study was a different condition (e.g., diabetic retinopathy (DR), diabetic nephropathy (DN)). After a first screening round, a subset of 185 studies was independently reviewed by two researchers, ensuring the thoroughness and validity of our results.

### Data Extraction

The main outcome of the meta-analysis was a list of proteins from each study, including FC and *p*-values, which were extracted directly from the articles. Additional data were also collected, including publication details, study design, proteomic methods, statistical analysis, and group information (demographic and clinical variables). Study investigators were contacted to obtain unreported primary outcomes. For all other variables, missing data directly impacted study quality in the risk of bias assessment. Some articles analyzed both proteomic and phosphoproteomic or glycoproteomic data; however, only proteomic data were included in this systematic review.

### Quality Assessment

QUADOMICS tool,^49^ adapted from the QUADS tool for omics-based studies, was used to assess the quality and risk of bias in the included studies (Supplementary Table S4).

### Data Processing

Due to inconsistent protein naming conventions across studies, we recognized the need for standardization and made this a transparent part of our research process. UniProt identifiers were used to enhance comparability across studies due to the ambiguity and variability of protein names. For articles that did not report UniProt identifiers, protein names were mapped to UniProt entries using the UniProt web-based mapping tool.^50^ Proteins without the corresponding UniProt identifier were excluded from the analysis. Since each protein identifier had to be unique within each study, an aggregation method based on the average FC and *p*-values was applied for each unique UniProt ID in two studies. For some studies, reanalysis of raw data was necessary due to the absence of results for our target groups, either fully or partially. When downstream analysis information was available, this process was reproduced accordingly. Details of the reanalysis procedures are fully described in Supplementary Table S5.

### Qualitative Assessment

To achieve a global understanding of the results, interaction networks between pairs of interesting proteins were generated using the STRING database,^51^ considering only strong interactions (score >0.7). Overrepresentation analysis (ORA) was performed using Gene Ontology (GO) databases to interpret the results at a functional level,^52,53^ followed by semantic clustering of the functional terms.^54^ A vote-counting analysis was computed for each shared protein, considering its direction of change for differential abundance between T2D and control groups.^46^

### Omics-Based Meta-Analysis

Protein outcomes (FC, *p*-values) were provided in some studies as adjusted *p*-values or log_2_-transformed FC (log_2_ FC). A general *p*-value adjustment was applied to data sets that only provided raw *p*-values, using the Benjamini–Hochberg correction and the total number of proteins involved in the comparison (retrieved from the corresponding articles). Both FC and log_2_ FC were estimated.

Proteins identified in at least two studies were selected, and their FC and adjusted *p*-values were aggregated, considering the group size using a weighted *p*-value combination as a variant of Fisher’s method and a weighted average of the log_2_ FC, respectively. Both approaches are implemented in the Amanida R package.^46^

The contribution of each study was determined by calculating the percentage of proteins from that study relative to the total number of proteins involved in the aggregation. To assess the influence of individual studies on the results of this omics-based meta-analysis, an influence analysis was performed: data were reanalyzed repeatedly, each time excluding one study, followed by the calculation of the Mean Absolute Percentage Error (MAPE) between the new FC and the original FC (previously estimated using all studies). This calculation was performed only for the subset of proteins included in each reanalysis. Higher MAPE values indicate greater differences between the aggregated log_2_ FC from the full meta-analysis and those from the leave-one-out study subsets.

### Random-Effects Meta-Analysis

Only studies that provided raw data were involved in the random-effects meta-analysis. Given the large number of proteins shared by at least two studies, only a subset of relevant and frequently reported proteins was analyzed. To select these, a list of relevant proteins explicitly mentioned in the included studies was compiled (Supplementary Table S6). Proteins from this list that were shared by more than two-thirds of the studies were selected for the meta-analysis.

Standardized Mean Difference (SMD) was selected as the effect size measure.^55^ A random-effects model with restricted maximum likelihood estimation was fitted to obtain the pooled SMD to explore potential differences between the T2D and control groups for each protein.^56^ Confidence intervals (CIs) (95%) for the pooled effect and individual measures were estimated. The heterogeneity between studies was assessed by τ^2^ and Higgins’ *I* ^2^ statistic,^57^ representing how much of the total variance could be attributed to the total amount of heterogeneity, with values ranging from 0 (all heterogeneity due to sampling variance) to 100 (all the variability due to heterogeneity between clusters). The goodness of fit was evaluated by sensitivity analyses based on outlier and influential case diagnostics plots (standardized residuals, hat values, and Cook’s distance).^58^ The assessment of publication bias was evaluated through contour-enhanced funnel plots.^59^ These plots show areas of statistical significance on a funnel plot (contours representing conventional levels of statistical significance: *p*-value <0.01, *p*-value <0.05, *p*-value <0.1), with effect sizes and standard errors on the two axes of the plot. Meta-analysis results are presented as a forest plot.

### Software

All analyses were performed using R software version 4.4.1 ^60^ with the following packages: *litsearchr*,^47^ *shiny*,^48^ *esc,* ^55^ *meta,* ^56^ *rbioapi,* ^51^ *clusterProfiler,* ^52^ *AnnotationDbi,* ^53^ and *GOSemSim.* ^54^ All codes are available in the GitHub repository for this systematic review: https://github.com/juliagcurras/metaanalysisT2D.

## Results

### Search Results

Refinement of the search equation increased the total number of retrieved articles from 969 in the initial search to 2,422 articles in the final search across the three databases (Supplementary Table S7). After removing duplicates based on DOI, 1,273 unique records remained for initial screening, with 505 articles identified as overlapping across the three databases (Supplementary Figure S1). Subsequently, 185 articles underwent full peer review. Following eligibility criteria, 25 studies were finally selected, with a high interreviewer agreement (concordance index = 0.705, *p* < 0.001, Supplementary Table S8). Two additional studies were identified through reference snowballing, yielding a final total of 27 included articles. The complete selection workflow is summarized in the PRISMA diagram (Figure 1), and the main characteristics of the 27 included studies are described in Table 1. A detailed description of the screening results and the final curated databases after data extraction is provided in Supporting Information 1.

**Figure 1:**
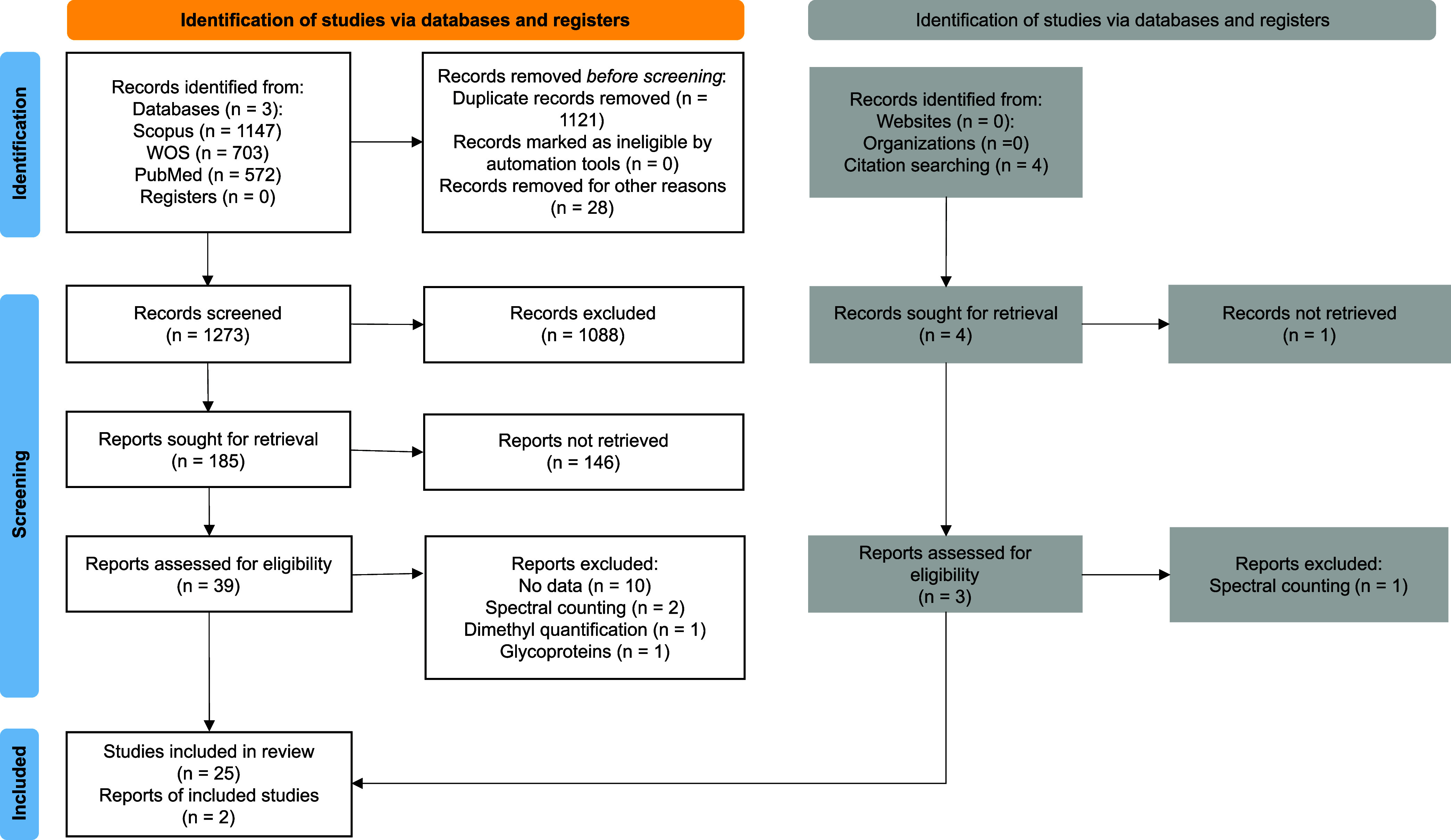
PRISMA workflow diagram.

**Table 1:** Description of the 27 Included Studies after the Search and Screening Process

Reference	Country	Year	Target disease?^a^	Mode	Type of sample	Sample size T2D	Sample size control	Raw data?
Li et al., 2018 ^61^	China	2018	Obesity	DDA	Plasma	10	9	Yes
Zhao et al., 2021 ^62^	China	2021	T2D	DDA	Plasma	8	8	No
Yu et al., 2022 ^63^	China	2022	MCI	DDA	Plasma	25	30	No
Kaur et al., 2012 ^64^	USA	2012	T2D	DDA	Serum	10	10	No
Abdulwahab et al., 2019 ^65^	Bahrain	2019	T2D	DIA	Serum	6	6	No
Chen et al., 2020 ^66^	China	2020	MTS	DDA	Serum	7	7	No
Nimer et al., 2023 ^67^	Jordan	2023	T2D	DIA	Serum	7	7	No
Darmayanti et al., 2023 ^68^	Indonesia	2023	DN	DDA	Serum	55	32	No
Li et al., 2023 ^69^	China	2023	T2D	DDA	Serum	9	9	Yes
Vestad et al., 2021 ^70^	Norway	2021	HIV	DDA	Extracellular vesicles (blood)	9	7	Yes
Nunez Lopez et al., 2022 ^71^	USA	2022	T2D	DDA	Extracellular vesicles (blood)	10	10	No
Skeie et al., 2018 ^72^	USA	2018	T2D	DDA	Endothelium–Descemet membrane (eye)	5	4	Yes
Zou et al., 2020 ^73^	China	2020	Dry Eye	DDA	Tear (eye)	10	10	Yes
Amorim et al., 2022 ^74^	Portugal	2022	DR	DDA	Tear (eye)	8	8	Yes
Sachdeva et al., 2024 ^75^	USA	2024	T2D	DDA	Aqueous humor (eye)	11	15	Yes
Lewandowicz et al., 2015 ^76^	Poland	2015	DN	DDA	Urine	15	12	No
Chen et al., 2021 ^77^	China	2021	DVD	DDA	Urine	22	21	Yes
Yan et al., 2024 ^78^	China	2024	DN	DIA	Urine	12	12	Yes
Thingholm et al., 2011 ^79^	Denmark	2011	T2D	DDA	Muscle biopses—myotubes	10	10	No
Chae et al., 2018 ^80^	South Korea	2018	T2D	DDA	Muscle biopses—mitochondria	9	9	No
Ferreira da Silva et al., 2024 ^81^	Brazil	2024	T2D	DIA	Saliva;Plasma	11	29	No
Samodova et al., 2025 ^82^	Denmark	2025	T2D	DIA	Saliva	15	15	No
Kim et al., 2014 ^83^	South Korea	2013	T2D	DDA	Visceral adipose tissue	5	6	No
Carruthers et al., 2021 ^84^	USA	2021	T2D	DDA	Visceral adipose tissue	10	10	No
Zhao et al., 2024 ^85^	China	2024	T2D	DDA	Liver tissue	3	3	Yes
Wigger et al., 2021 ^86^	Germany	2021	T2D	DDA	Pancreatic island cells	5	5	Yes
An et al., 2018 ^87^	China	2018	T2D	DDA	Sperm	6	6	Yes

a HIV: Human immunodeficiency virus; DN: diabetic nephropathy; DVD: diabetic vascular dementia; DR: diabetic retinopathy; MCI: mild cognitive impairment; MTS: metabolic syndromes.

### Quality Assessment

A structured quality assessment of the selected studies was conducted based on the QUADOMICS framework (Supporting Information 2). According to this evaluation, 12 studies were classified as high quality (more than 50% positive answers); 8 as low quality (more than 50% negative responses or more negative than positive responses); and the remaining studies as moderate quality due to a high proportion of unclear responses (Figure 2A).

**Figure 2:**
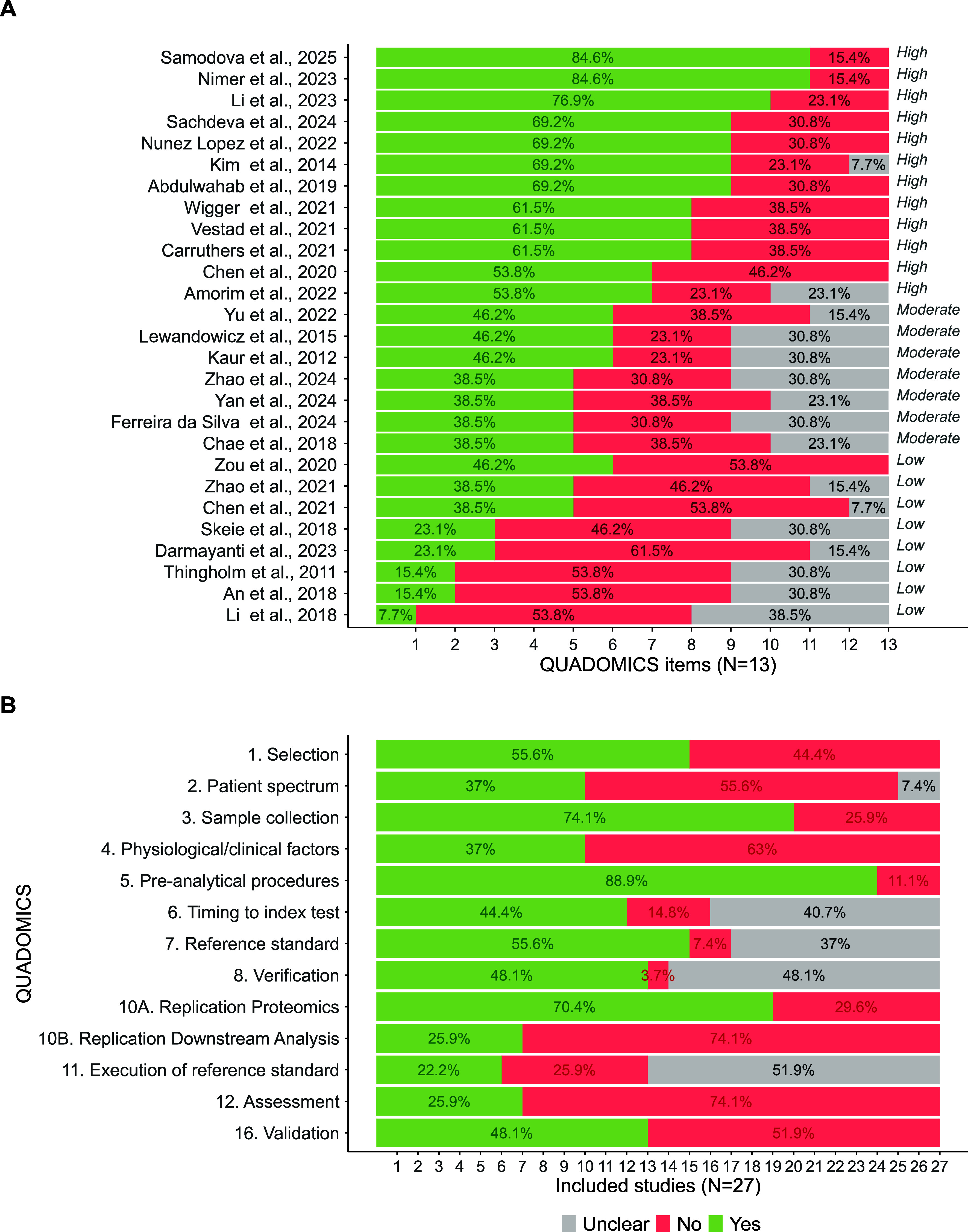
Quality assessment of the included studies using the QUADOMICS tool. (A) Quality profile for each included study, with quality assignments (high, moderate, low). (B) Proportion of studies fulfilling each QUADOMICS item, including those with positive, negative, and unclear responses.

As shown in Figure 2B, approximately half of the articles (55.6%) reported inclusion and exclusion criteria (item 1) followed in the recruitment process. Only 37% of the final studies fulfilled the criterion of having no age/sex restrictions and an adequate patient source (item 2), which can be partially explained by some articles targeting conditions related to, but distinct from, T2D. For example, Yu et al., 2022 ^63^ investigated the role of T2D in mild cognitive impairment using elderly patients, who are not an ideal patient source to assess T2D specifically. Detailed information about the sample type and collection procedures (item 3) was reported in 74.1% of the studies, especially for unusual sample types (semen, visceral adipose tissue, or aqueous humor). However, most articles failed to report the timing of sample collection and storage intervals.

Regarding demographic and clinical factors (item 4) for both T2D and control groups, only 37% of studies provided sufficient information, with diabetes duration being the least reported variable. Reporting tended to be more detailed for case groups than for controls. In terms of analytical bias, over 70% of the studies supplied adequate details on preanalytical procedures and proteomic analysis (items 5 and 10A). However, only 25.9% provided enough reproducible descriptions of downstream analysis (item 10B), often lacking key information on data normalization, data imputation, and *p*-value adjustments. Unclear responses were most frequent for items related to the index test, particularly the diagnostic criteria for T2D (item 7), timing of diagnosis (item 6), condition verification (item 8), and diagnostic implementation (item 9), all of which were poorly documented.

Finally, only 25.9% of studies conducted discrimination analysis of the relevant biomarkers (item 12), while biomarker validation using alternative techniques such as Western blot or ELISA was reported in nearly half of the studies (item 16, 48.1%).

### General Description of Relevant Literature

A total of 27 final articles met the inclusion criteria, most of which were conducted in China (37.0%) and the United States (18.5%). These studies span from 2011 to 2025, with a notable increase in publications after 2018 (Supplementary Figure S2A–B). Eight different sample types were used, with serum (21.4%), eye tissue (14.3%), and plasma (14.3%) being the most frequently analyzed (Supplementary Figure S2C). Only one study (Ferreira da Silva et al., 2024 ^81^) analyzed two different sample types (plasma and saliva) from the same cohort.

Regarding the proteomic analyses, 81.5% of the studies operated in the DDA mode. Instruments from Thermo commercial houses (including Thermo Fisher Scientific, Thermo Scientific, and Thermo Electron) were used in 70.3% of the studies (Supplementary Figure S2D). UniProt was the most commonly referenced protein identification library, cited in 63.0% of the articles. MaxQuant was the most frequently used software for protein identification (29.6%), followed by Proteome Discoverer (18.5%). DAA was commonly conducted using Perseus and *R* (18.5% each).

In terms of statistical analysis, testing was heterogeneous across the studies. The *t*-test was used in 44.4% of the studies, and functional analysis was performed in nearly all studies (88.9%). However, several gaps were identified in the reporting of downstream analytical procedures: 18.5% of studies did not report the analysis software used; 40.7% did not specify the normalization method; 44.4% did not indicate *p*-value adjustment procedures; and 63.0% lacked information on data imputation. Detailed settings are shown in Supplementary Table S9.

As highlighted in the QUADOMICS section, group-level demographic and clinical data were frequently incomplete, particularly for control groups. Age and gender information were generally provided, but important factors related to diabetes—such as BMI (Body Mass Index), HbA1c, and FPG (Fasting Plasma Glucose)—were not reported in 44.4% to 62.9% of the articles (Supporting Information 2). Group descriptions show that, in most articles, the average age of the T2D and control groups is similar, except for Kaur et al., 2012,^64^ Ferreira da Silva et al., 2024,^81^ and Kim et al., 2014,^83^ where the control groups were younger. Participants across all studies ranged in age from approximately 20 to 80 years old (Supplementary Figure S3A). Gender distribution was generally balanced between T2D and control groups (Supplementary Figure S3B). The female percentage across studies ranged from 30% to 60%, with two articles including only men (An et al., 2018;^87^ Darmayanti et al., 2023 ^68^—where the latter focused on semen samples) and one study including only women (Carruthers et al., 2021 ^84^). Due to the well-established relationship between obesity and diabetes, BMI data highlighted those studies including obese patients; however, for most studies, BMI ranged from 20 to 30 (Supplementary Figure S3C). Forest plots of average HbA1c and FPG levels (Supplementary Figure S3D–E) confirmed group assignment in studies reporting this information. T2D duration was reported in only nine studies. In two cases, patients were diagnosed at recruitment (duration = 0 years), while the remaining studies reported durations ranging from 3 to 12 years, with substantial intra- and interstudy variability (Supplementary Figure S3F).

### Qualitative Analysis

From the 27 included studies, a total of 23,099 protein identifiers were initially retrieved. After data curation and harmonization, this number was reduced to 22,328. Among these, 2,745 proteins were reported in ≥2 studies and 85 were consistently identified in at least eight articles (Supplementary Figure S4A). This highly recurrent subset of proteins showed strong interconnections, as illustrated by the interaction network in Figure 3A (Supporting Information 3).

**Figure 3:**
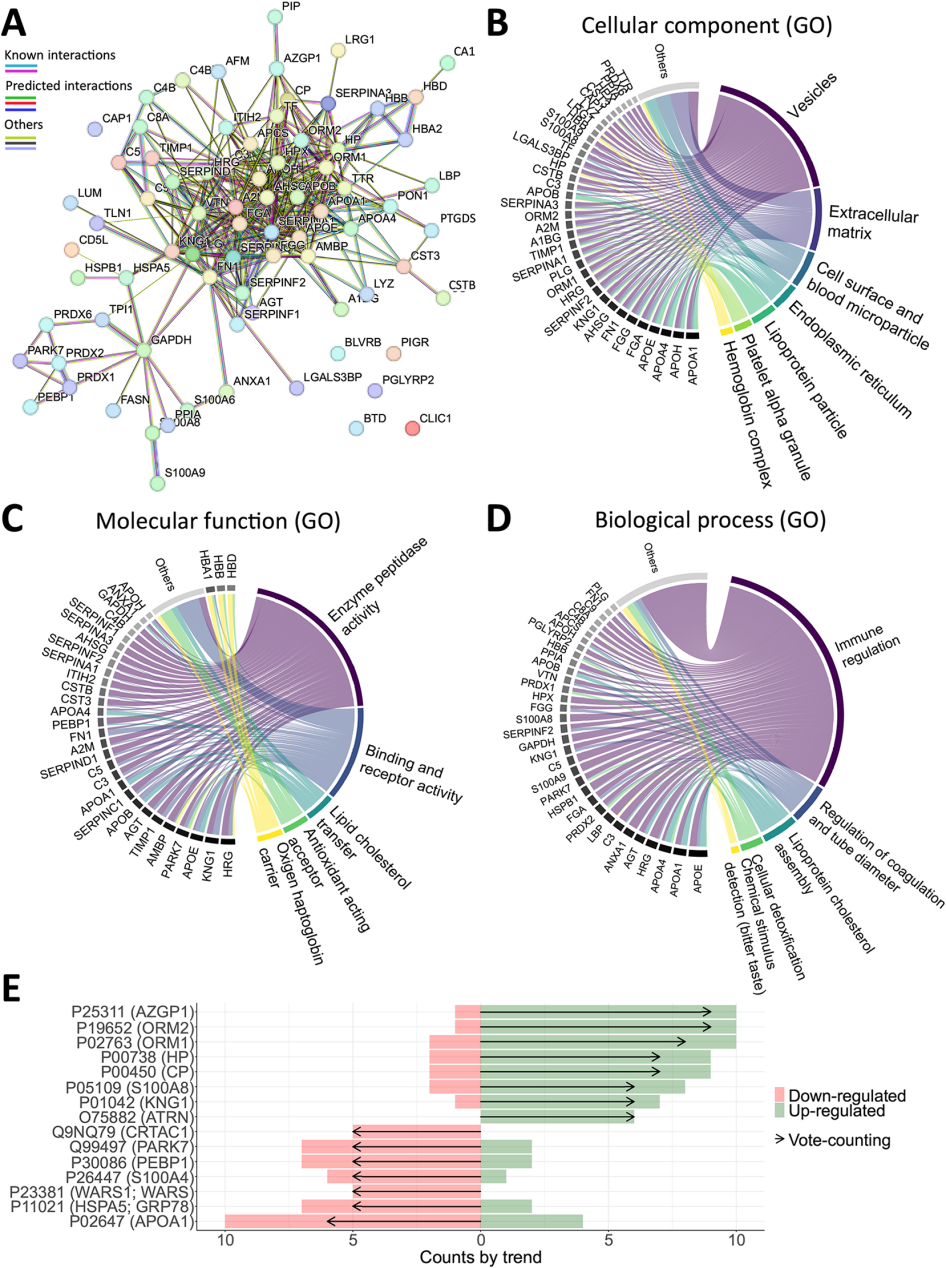
Qualitative analysis of proteins commonly analyzed in proteomic studies of T2D. (A) Protein–protein interaction network (score >0.7) of the proteins identified in at least eight studies (*n* = 85). Edge color code, in legend order: known interactions from curated databases or experimentally determined; predicted interactions by gene neighborhood, gene fusions, or gene cooccurrence; others include text mining, coexpression, and protein homology. (B–D) Circle plot showing the association and contribution of proteins to functional annotation clusters for cellular components (B), molecular function (C), and biological process (D) of Gene Ontology (GO). Proteins linked to only a few terms are grouped under the *Others* class. (E) Vote-counting analysis summarizing the direction of differential abundance (upregulated or downregulated in T2D) across studies for selected proteins.

ORA was performed on these 85 proteins, using the broader set of 2,745 proteins (shared by at least two studies) as the reference background. Statistically significant enrichment terms (adjusted *p*-value and *q*-value <0.05) were divided by GO category and grouped by semantic clustering. The clustering results for the 30 significant cellular component annotations indicate that the 85 proteins are mainly localized in lipoprotein particles, the extracellular matrix, various types of vesicles (cytoplasmic, secretory, luminal), the endoplasmic reticulum, the cell surface, and blood-related complexes and particles (Figure 3B). Regarding the main clusters for the 38 significant molecular functional terms, enrichment indicates associations with lipid and cholesterol transfer, binding and receptor activity (heparin, protease, oxygen, hemoglobin, haptoglobin), as well as enzyme peptidase activity, antioxidant-acting acceptors, and oxygen/haptoglobin carriers (Figure 3C). In terms of biological processes (114 after simplifying by removing redundancies), the major clusters refer to proteins that are primarily involved in immune regulation—including complement activation and humoral and innate immunity—as well as lipoprotein cholesterol assembly, cellular detoxification, regulation of coagulation and tube diameter, and chemical stimulus, particularly for the detection of bitter taste (Figure 3D). The complete ORA results and the clustering of functional terms can be accessed in Supporting Information 3.

Complementing this analysis, a vote-counting approach was applied to assess the directionality of protein regulation based on log_2_ FC. This analysis revealed that zinc-alpha-2-glycoprotein (*AZGP1*) and myosin regulatory light chain 1 (*ORM2*) were consistently upregulated in the T2D group across most studies, whereas apolipoprotein A-I (*APOA1*) was generally downregulated in the T2D group (Figure 3E, Supporting Information 3).

### Omics-Based Meta-Analysis

Of the 27 included studies, 23 provided full quantitative data reporting both FC and *p*-values for each protein (three lacked specific *p*-value information, and one only provided the direction of change). These studies were included in the omics-based meta-analysis. From a total of 16,668 proteins reported across these 23 articles, 2,735 were found to be shared by at least two studies (Supplementary Figure S4B). Aggregated log_2_ FC values and adjusted *p*-values for these proteins are presented in Figure 4A, with full details available in Supporting Information 3.

**Figure 4:**
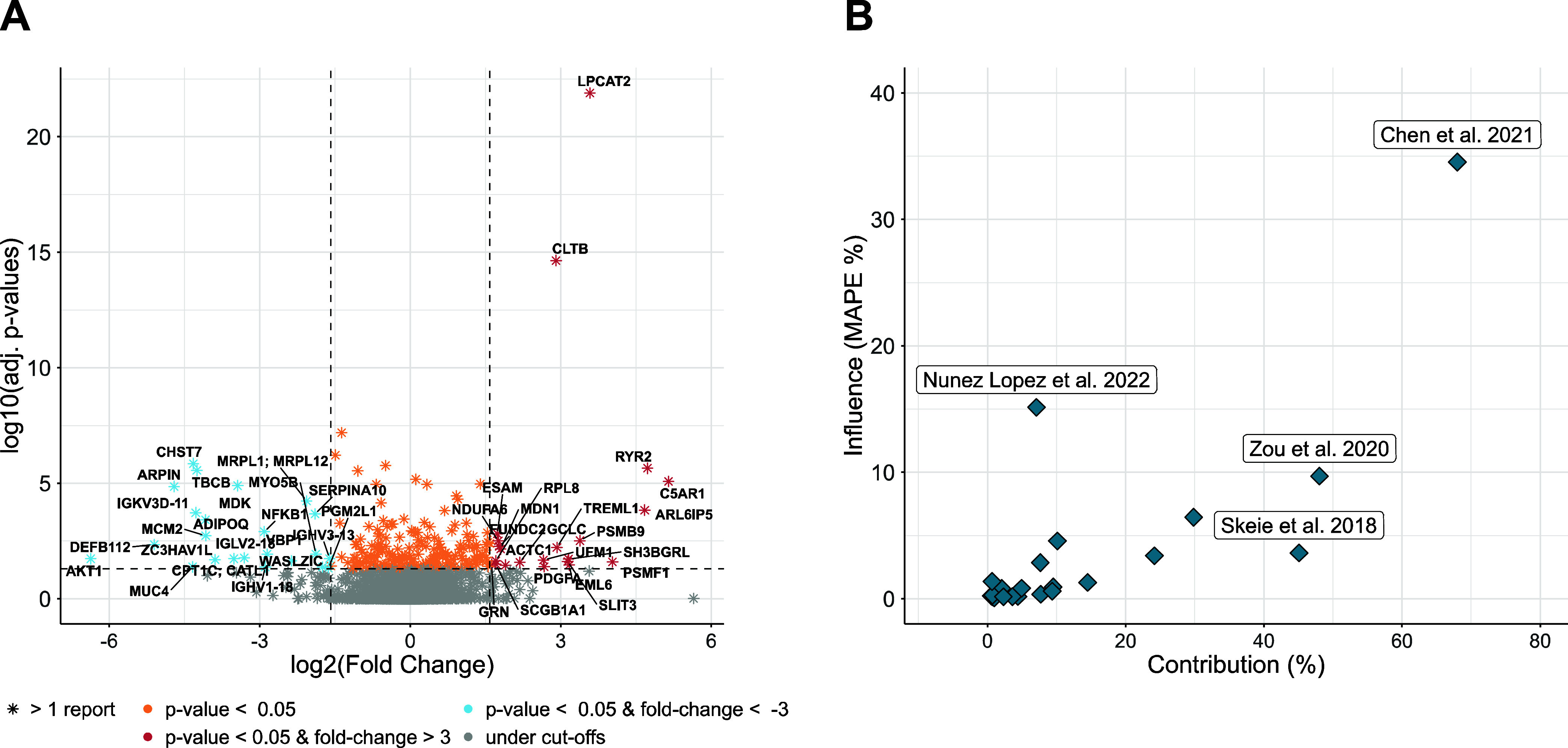
(A) Volcano plot showing the aggregated adjusted *p*-values and log_2_ FC, weighted by sample size, for the 2,734 proteins shared by two or more studies. Positive log_2_ FC values indicate higher protein abundance in the T2D group. (B) Correlation between contribution and influence (MAPE %) of the included studies.

Contribution analysis revealed that three studies—Chen et al., 2021,^77^ Zou et al., 2020,^73^ and Skeie et al., 2018 ^72^—collectively accounted for more than 40% of the proteins shared across studies. However, influence analysis, which evaluates the impact of each study on the meta-analytic results, identified Chen et al., 2021,^77^ Nunez Lopez et al., 2022,^71^ and Zou et al., 2020 ^73^ as the most influential contributors. Chen et al., 2021 ^77^ showed the highest influence: when this study was omitted, the pooled FC values differed by 34.5% relative to the pooled estimates obtained from all 23 studies. The impact of Nunez López et al., 2022 ^71^ and Zou et al., 2020 ^73^ was considerably lower, but also important (15.1% and 9.7%, respectively). For the remaining studies, influence values ranged from 0.07% to 6.44%, with 13 studies showing values below 1%. Interestingly, despite its large protein count, Skeie et al., 2018 ^72^ was not found to be highly influential (3.63%). This highlights that while contribution and influence are related, a high volume of data does not necessarily equate to a strong impact on meta-analytic outcomes (Figure 4B, Supporting Information 3).

Once the aggregated FC and adjusted *p*-values were computed, proteins were filtered to identify relevant, stable biomarkers shared across studies while minimizing the influence of previously identified high-impact studies. Criteria included an aggregated adjusted *p*-value <0.05, an absolute log_2_ FC > 1, and presence in three or more studies. A subset of 29 significant proteins met these thresholds. Further filtering resulted in the identification of seven stable candidate biomarkers for T2D (Table 2). The filter was based on consistency in the log_2_ FC direction across studies and exclusion of results heavily driven by high-influence studies (Figure 5), excluding the most influential study. Briefly, these seven candidates can be grouped as follows:

- Four related to metabolic regulation: Vesicular integral-membrane protein VIP36 (*LMAN2*), truncated apolipoprotein A-II (*APOA2*), saposin-B-Val (*PSAP*), and dihydrolipoyl dehydrogenase (*DLD*).
- Two involved in cell binding and adhesion: Collagen alpha-1­(VI) chain (*COL6A1*) and cadherin-1 (*CDH1*).
- One associated with regulation of transcription and mitochondrial function: Prohibitin (*PHB1*).

**Figure 5:**
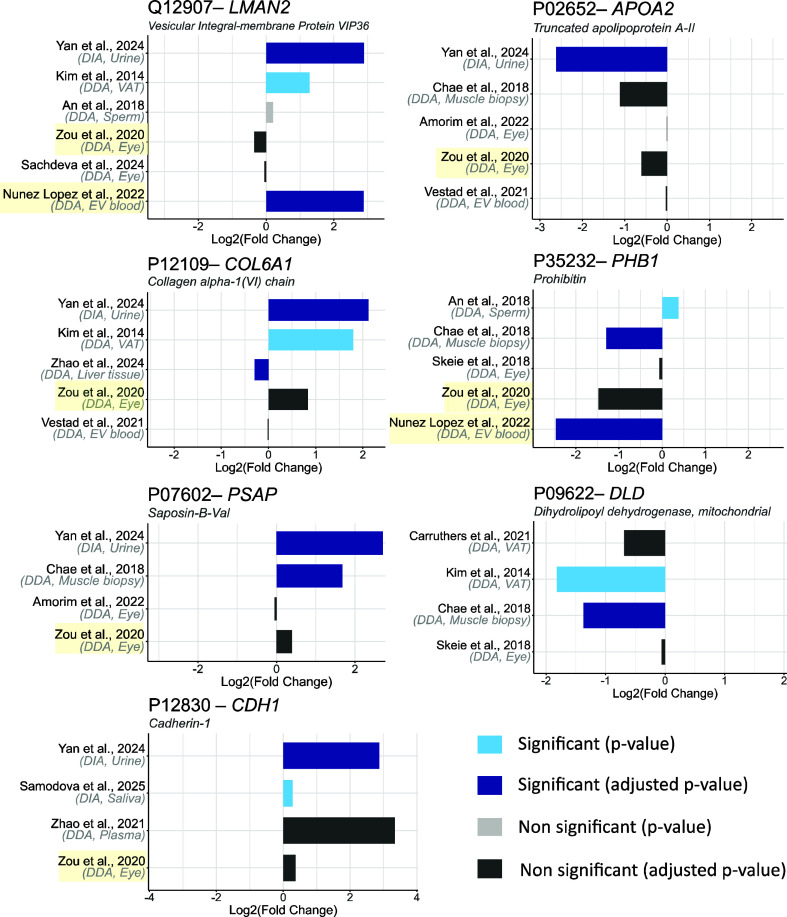
Log_2_ FC magnitude and direction for the filtered proteins in each of the studies, with bars colored by original significance interpretation. Most influential studies are highlighted in yellow. Positive log_2_ FC values indicate higher protein abundance in the T2D group. EV: extracellular vesicles; VAT: visceral adipose tissue.

**Table 2:** Summary of Seven Key Protein Biomarkers Identified through Omics-Based Meta-Analysis of High-Throughput Proteomic Studies Comparing T2D and Normoglycemic Individuals

UniProt ID	Protein name	Gene symbol	Adj. *p*-value	Log_2_ FC	GO annotations^a^	(No. studies) References
**Q12907**	Vesicular integral membrane protein (VIP36)	*LMAN2*	0.041	1.196	Protein binding (GO:0005515)	(6) Nunez Lopez et al., 2022;^71^ Kim et al., 2014;^83^ Yan et al., 2024;^78^ Sachdeva et al., 2024;^75^ Zou et al., 2020;^73^ An et al., 2018 ^87^
					Carbohydrate binding (GO:0030246)	
					D-mannose binding (GO:000557)	
**P02652**	Truncated apolipoprotein A-II	*APOA2*	0.048	–1.017	Cholesterol homeostasis (GO:0042632)	(5) Chae et al., 2018;^80^ Yan et al., 2024;^78^ Vestad et al., 2021;^70^ Zou et al., 2020;^73^ Amorim et al., 2022 ^74^
					Response to glucose (GO:0009749)	
					Protein oxidation (GO:0018158) and stabilization (GO:0050821)	
**P12109**	Collagen alpha-1(VI) chain	*COL6A1*	0.023	1.108	Protein binding (GO:0005515)	(5) Kim et al., 2014;^83^ Zhao et al., 2024;^62^ Yan et al., 2024;^78^ Vestad et al., 2021;^70^ Zou et al., 2020 ^73^
					Collagen binding (GO:0005518)	
					Platelet-derived growth factor binding (GO:0048407)	
**P35232**	Prohibitin	*PHB1*	0.027	–1.244	Regulation of DNA-templated transcription (GO:0006355)	(5) Chae et al., 2018;^80^ Nunez Lopez et al., 2022;^71^ Zou et al., 2020;^73^ Skeie et al., 2018;^72^ An et al., 2018 ^87^
					Positive regulation of complement activation (GO:0045917)	
					Antiviral innate immune response (GO:0140374)	
					Mitochondrion organization (GO:0007005)	
**P07602**	Saposin-B-Val	*PSAP*	0.004	1.314	Positive regulation of β-galactosidase activity (GO:1903771)	(4) Chae et al., 2018;^80^ Yan et al., 2024;^78^ Zou et al., 2020;^73^ Amorim et al., 2022 ^74^
					Lysosomal transport (GO:0007041)	
					Ganglioside GM1 transport to membrane (GO:1905572)	
**P09622**	Dihydrolipoyl dehydrogenase, mitochondrial	*DLD*	0.038	–1.019	Dihydrolipoyl dehydrogenase (NADH) activity (GO:0004148)	(4) Chae et al., 2018;^80^ Kim et al., 2014;^83^ Carruthers et al., 2021;^84^ Skeie et al., 2018 ^72^
					Protein binding (GO:0005515)	
**P12830**	Cadherin-1	*CDH1*	0.001	1.534	Positive regulation of protein localization (GO:1903829)	(4) Samodova et al., 2025;^82^ Zhao et al., 2021;^62^ Yan et al., 2024;^78^ Zou et al., 2020 ^73^
					Cell–cell adhesion (GO:1903829)	
					Regulation of gene expression (GO:0010468)	
					Positive regulation DNA-templated transcription (GO:0045893)	

a GO annotations associated with each protein correspond to the most reliable ECO (Evidence and Conclusion Ontology) terms inferred from the experiment, direct assay, mutant phenotype, and genetic or physical interaction.

Several of the previously identified biomarkers are also associated with key biological processes relevant to T2D, including immune response, cholesterol homeostasis, and carbohydrate metabolism.

A grouped analysis was also conducted by sample type, limited to those samples analyzed in more than one study (Supplementary Figure S5). In general, no relevant biomarkers were identified in blood (serum and plasma) or eye samples, and those found in other sample types were each supported by a single study, providing weak evidence of their relevance. Urine, however, was an exception, with aggregated results showing multiple significant proteins and considerable magnitudes of change. Nevertheless, this evidence may be unreliable, as it is primarily supported by the most influential study in the global meta-analysis: Chen et al., 2021.^77^

### Random-Effects Meta-Analysis

Twelve articles provided proteomic raw data and were included in the random-effects meta-analysis. Due to the large number of proteins reported across studies, a selection process was implemented as described in the Methods section to focus on the most consistently identified proteins. The final list comprised highly shared proteins across multiple studies, including alpha-1-acid glycoprotein 1 (*ORM1*), reported in 10 studies; alpha-1-antichymotrypsin (*SERPINA3*) and truncated apolipoprotein A-I (*APOA1*), both present in 9 articles; and six proteins identified in 8 studies: Haptoglobin (*HP*), apolipoprotein E (*APOE*), vitronectin (*VTN*), zinc-alpha-2-glycoprotein (*AZGP1*), peroxiredoxin-2 (*PRDX2*), and peptidyl-prolyl cis–trans isomerase A (*PPIA*) (Supplementary Table S10).

Results from the random-effects meta-analysis revealed limited consistency in the SMD values across studies for the most of these proteins (Supplementary Figure S6). However, three proteins—*ORM1*, *HP*, and *AZGP1*—showed consistent differences (although without reaching significance) in abundance between T2D and control groups (Figure 6). All three proteins were upregulated in the T2D group, with *AZGP1* displaying the highest effect size (SMD (95% CI) = 0.78 (−0.13 to 1.69)).

**Figure 6:**
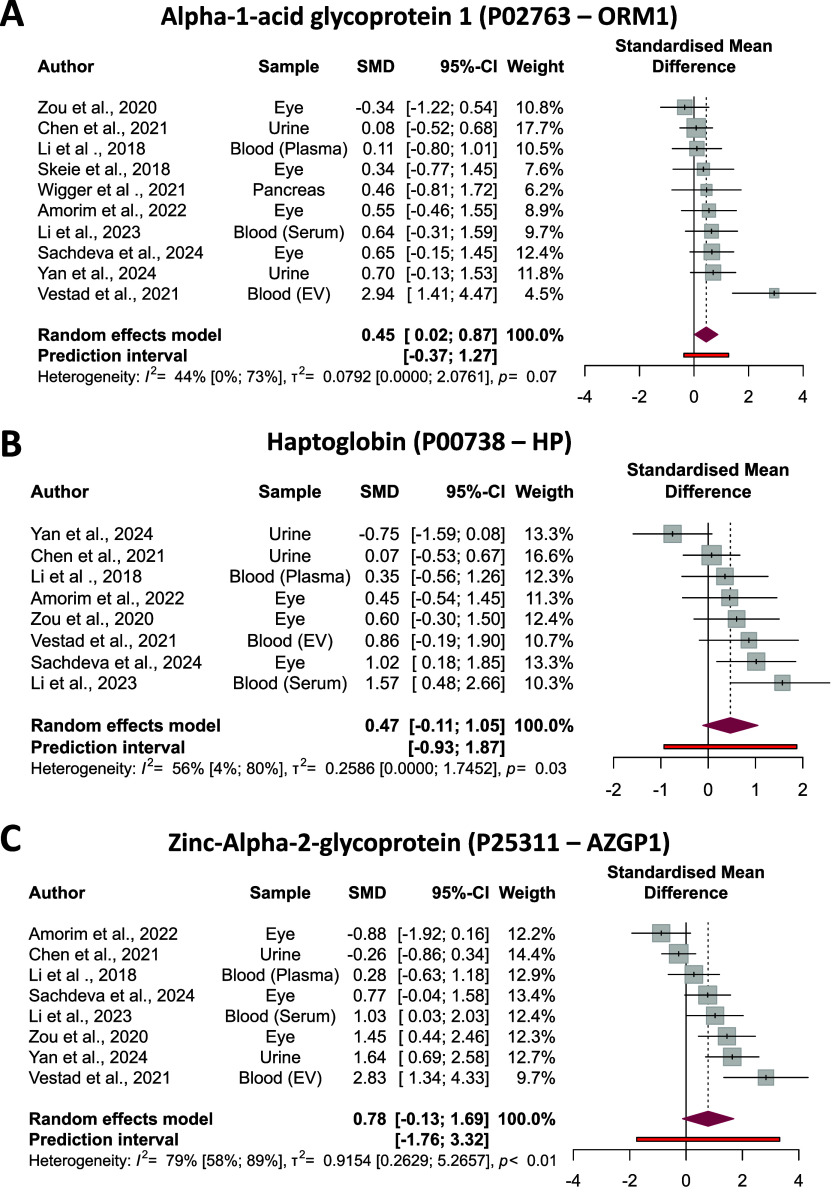
Forest plot displaying the SMDs for selected proteins across studies comparing T2D and normoglycemic individuals. Individual study effect sizes, 95% confidence intervals, and corresponding weights are shown alongside the pooled SMD estimated using a random-effects model. Measures of heterogeneity—including *I* ^2^ (percentage of total variability due to heterogeneity), τ^2^ (between-study variance), and the *p*-value from Cochran’s *Q* test—are reported to assess consistency across studies. Positive SMD values indicate a higher protein abundance in the T2D group.

To assess the robustness of the meta-analyses for the previous three proteins, sensitivity, bias, and influence diagnostics were performed. One outlier study (Vestad et al., 2021 ^70^) was identified for *ORM1*, and its removal substantially reduced heterogeneity (*I* ^2^ from 44% to 0%). No outliers were detected for *HP* or *AZGP1*. Egger’s test suggested no significant small-study effects for any protein (all *p* > 0.05), though a trend toward asymmetry was observed for *ORM1* (*p* = 0.065). Trim-and-fill analysis did not impute missing studies for *ORM1* but indicated potential asymmetry and increased heterogeneity for *HP* and *AZGP1*. Excess significance testing showed no inflation for *ORM1* or *HP*, while *AZGP1* exhibited a borderline significant excess (*p* = 0.048). Selection models detected possible publication bias only for *ORM1* (*p* = 0.033). Influence analysis confirmed that pooled estimates were stable across studies, with no single study unduly altering the results. Overall, findings appeared robust, though caution is warranted for *ORM1* due to potential bias indicators. Detailed results from outlier detection, publication bias, and influence diagnostics are presented in Supplementary Tables S11 to S14.

Due to the high heterogeneity observed in the meta-analysis, we explored potential sources of variability for each consistent biomarker (Supplementary Table S15). *ORM1* and *AZGP1* did not show significant differences across subgroups defined by sample type, data acquisition method, study quality, commercial platform, or analytical software. In contrast, all of these factors appear to contribute to variability for *HP*, which exhibited, for example, higher SMD values in urine samples and in studies rated as low quality according to QUADOMICS reporting criteria.

### metaMarkers2D

To enhance the accessibility and impact of this study for the scientific community, a Shiny app—*metaMarkersT2D*—was developed. This interactive tool enables users to explore the final curated database of proteins identified in the 27 studies included in the meta-analysis. Proteins can be searched using their UniProt ID, which serves as a standardized reference identifier.

Upon querying a protein, the application returns a list of studies in which that protein was reported, including the original FC and *p*-value or adjusted *p*-value for each occurrence. To provide further context, additional metadata about the studies is organized into three separate tables, offering detailed information about experimental design, sample types, and analysis methods.

Differential abundance results are visualized in a bar plot, where the *Y*-axis represents the magnitude of the log_2_ FC and the *X*-axis lists the studies. Bars are color-coded according to the significance level reported in the original articles (with *p*-values or adjusted *p*-values specified). For ease of use, all retrieved data can be downloaded in a tabular format. The app is freely accessible at the following link: https://jgcurras.shinyapps.io/metaMarkersT2D/.

## Discussion

In this study, we conducted a systematic review of proteomic biomarkers associated with T2D. We integrated quantitative data from the selected studies using both an omics-based meta-analysis and a traditional random-effects meta-analysis. This combined approach enabled us to identify key protein biomarkers that were consistently reported across studies with concordant directions of change, statistical significance, and clear biological relevance to T2D pathophysiology.

Our final set of 27 articles clearly reflects that T2D has been studied from multiple perspectives, focusing mainly on noninvasive or minimally invasive samples such as blood, urine, eye tissue, and even semen, while also including primary tissues directly involved or affected by impaired glucose homeostasis: liver, pancreas, muscle, and visceral adipose tissue. This diversity of tissues can also be explained by the fact that in some studies, the primary target was not T2D itself but microvascular complications derived from it, particularly DN and RN. In studies reporting cohort details, T2D and control groups were generally well matched for sex, age, and BMI, with only moderate variability. Because obesity is strongly associated with T2D,^88^ it could have been a confounding factor; however, we mitigated this by including only studies in which both T2D and control groups had similar obesity status.

Details of the proteomic analyses are generally provided, revealing substantial variability in instrument platforms and settings across studies. In contrast, the reporting of critical data processing steps (normalization, missing-data imputation, and statistical testing) was often lacking. Although DIA methods offer clear advantages in proteomics, few studies have used this approach, likely due to its relatively recent introduction. On a positive note, the use of the same reference library, UniProt database, for protein identification in most studies likely contributed to greater consistency in the proteins analyzed. Based on the extraction and analysis of baseline data from the included studies, we recommend that future work should pay particular attention to detailed reporting of case and control group descriptions, diagnostic methods, disease duration, and recruitment processes. Such information is essential to assess comparability across studies and to ensure the representativeness of comparison groups, maximizing the value of each study’s data for meta-analytical approaches. Likewise, transparent reporting of data processing steps is critical since these have a substantial impact on the final results and are key to ensuring reproducibility.

Despite the variability introduced by different proteomic platforms, acquisition modes, sample types, and population origins, we identified a subset of 85 proteins reported in eight or more studies comparing T2D with the normoglycemic controls. These were not randomly shared proteins: their strong interaction networks and enriched functional annotations suggest their biological importance, with many processes representing the functional background of the general proteome commonly detected in differential abundance studies of T2D versus those of normoglycemic individuals. The main cellular locations of these highly recurrent proteins—including vesicles, extracellular matrix, cell surfaces, and circulating complexes or granules—underscore the relevance of interorgan communication in T2D pathophysiology. This is further supported by the enriched molecular functions related to binding and receptor activity, as well as peptidase activity, carried out by proteins such as fibronectin (*FN1*), histidine-rich glycoprotein (*HRG*), kininogen-1 (*KNG1*), alanine-glyoxylate aminotransferase (*AGT1*), complement factors (*C3*, *C5*, *C4B*), and proteins related to coagulation from the *SERPIN* genetic family. Collectively, these functions point to immune regulation (reflecting T2D’s chronic low-grade inflammation) and to coagulation and vascular tone control, processes closely linked to T2D micro- and macrovascular complications.^89^

In addition, several apolipoproteins such as apoliproteins E, B, A1, and 4 (*APOE*, *APOB*, *APOA1*, *APOA4*) were predominantly located in lipoprotein particles and were associated with lipid and cholesterol transport.^90^ Given that dyslipidemia often coexists with T2D and patients often exhibit an atherogenic lipid profile, alterations in lipoprotein particles are expected.^91^ A smaller subset of proteins, including *HP*, Parkinson’s disease protein 7 (*PARK7*), and hemoglobin subunits alpha 1, beta, and delta (*HBA1*, *HBB*, *HBD*), showed antioxidant and oxygen transport functions, highlighting the importance of cellular detoxification under the oxidative stress imposed by chronic hyperglycemia.^92^ We also observed enriched terms related to chemical stimulus detection, particularly bitter taste perception, involving proteins such as *AZGP1*, polymeric immunoglobulin receptor (*PIGR*), and prolactin-inducible protein (*PIP*).^93^ Altered flavor perception may influence dietary behavior and lifestyle, potentially contributing to metabolic disorders such as T2D.^94,95^ Notably, several of the previously mentioned proteins showed consistent regulation patterns across studies, with *AZPG1*, *HP*, and *KNG1* mostly upregulated, while *APOA1* and *PARK7* were predominantly downregulated.

Selecting relevant biomarkers based on statistical significance and magnitude of change proved challenging. In our omics-based meta-analysis, even widely shared proteins showed large discrepancies in adjusted *p*-values between studies, preventing many from meeting stringent significance thresholds in the aggregate analysis. These discrepancies were also reflected in the magnitude of change. As a result, the final biomarkers identified through this meta-analytic approach were reported in no more than six studies. Another key factor was the limited contribution of some studies to the shared protein set due to incomplete reporting of full or partial protein lists. In fact, blood-derived samples were the most frequently analyzed, raising the possibility of bias toward blood-associated proteins. However, these studies generally contributed shorter protein lists, which helped counteract potential overrepresentation effects. Despite these challenges, the omics-based meta-analysis proved to be a valuable strategy to address incomplete data reporting in proteomic studies by leveraging available fold-change and *p*-value metrics. In contrast, the more traditional random-effects meta-analysis could only include the 12 studies providing a raw quantitative matrix, underscoring the urgent need for open and standardized data sharing in omics research. Interestingly, this approach also identified multiple proteins reported in more than one article, but when a protein was present in four or more studies, concordant significance across data sets was rarely achieved, making it difficult to select a robust subset of biomarkers solely based on *p*-values. Consequently, the analysis was restricted to proteins previously reported as candidate biomarkers in the literature and supported by sufficient studies to strengthen the statistical power, regardless of significance. Combining both meta-analytic strategies, we identified ten candidate biomarkers supported by multiple studies as potential components of a multimarker diagnostic panel for T2D: *LMAN2*, *APOA2*, *PSAP*, *DLD*, *COL6A1*, *CDH1*, *PHB1, ORM1, HP*, and *AZPG1*.

From this list, some proteins were also identified as relevant biomarkers in previous reviews for T2D and proteomic biomarkers. All proteins found to be highly consistent in our random-effects meta-analysis had been reported previously in at least one of these reviews. *HP* was the most frequently mentioned, appearing in four reviews,^39,40,43,44^ three of which indicated higher *HP* levels in T2D,^40,43,44^ consistent with our findings. *AZGP1* was also widely reported,^43,44^ with previous reviews likewise describing increased abundance in T2D, further supporting our results. Additionally, our analysis identified *ORM1* as a promising biomarker, with high levels in T2D. This finding aligns with the review by Sohail et al., 2018,^40^ including concordance in the direction of change. Regarding the consistent biomarkers detected by the omics-based approach, two had been reported in prior studies. Various apolipoproteins were noted as relevant for T2D in the related literature,^39,43,44^ but *APOA2* was specifically highlighted by Riaz, 2015,^44^ who also indicated reduced abundance in T2D, consistent with our meta-analysis results. Furthermore, Riaz 2015 ^44^ also reported *CDH1* as a relevant biomarker for T2D, with increased concentrations in this condition, fully concordant with our findings.

Biomarkers highlighted in previous reviews were numerous and often variable. These lists essentially reflect collections of results from individual studies, with only a few studies supporting the relevance of each protein and almost no quantitative information provided. Moreover, the selection of articles was typically based on the authors’ discretion, introducing subjectivity and limiting reproducibility. In contrast, our systematic approach addresses these limitations by performing a structured and comprehensive search across three different databases to capture the relevant literature. Furthermore, the biomarkers identified in our study are accompanied by quantitative data on significance, direction, and magnitude of change, estimated through meta-analytical methods that appropriately account for variability between studies. Importantly, each of these biomarkers is supported by at least four studies, with some supported by up to ten, providing a robust and reproducible evidence base. Despite the strengths of this approach, several limitations should also be acknowledged in this work. One key issue is the lack of detailed preprocessing information and raw data in the included studies, which restricted our ability to perform uniform reanalyses and fully assess differential protein abundance. Variability in data processing methods, including normalization, imputation, and statistical testing procedures, is another important concern, which may have introduced additional heterogeneity and affected comparability across studies. In this regard, the random-effects meta-analysis is more robust than the omics-based approach, as it operates directly on the quantification matrices, thereby reducing the impact of differences in testing methods. A further limitation is the inconsistency in protein nomenclature, requiring extensive mapping to standard identifiers and potentially resulting in minor mismatches or omissions. As our analyses were conducted using previously published data sets, external validation of the candidate biomarkers was not feasible. This aspect should be addressed in future studies to provide additional validation of the findings.

In light of this work, we stress the importance of cautious interpretation of individual biomarker discovery studies and advocate for integrating evidence from previous work to corroborate or challenge proposed biomarkers, even qualitatively. We also emphasize the urgent need for greater data accessibility in omics research: adherence to FAIR (Findable, Accessible, Interoperable, Reusable) data principles should be mandatory for publication to ensure that data sets can be readily reused and reanalyzed by the scientific community.

Regarding T2D, future work should integrate proteomic data obtained using complementary technologies such as Olink and SOMAscan. For both integrated analyses and new biomarker studies, our metaMarkersT2D Shiny application developed in this study can serve as a useful tool to cross-reference novel findings against the database compiled in this review. Additionally, other omics layers, particularly lipidomics and metabolomics, which are closely linked to T2D pathogenesis, should be incorporated to build a more comprehensive multiomics profile of the disease. Systematic reviews and meta-analyses represent optimal approaches for this integration, as they minimize selection bias and generate interpretable quantitative metrics. In this context, the pubmedToCsv Shiny app developed in this project can assist in managing proteomic literature from the PMC database during the search step. Ultimately, pooling evidence from multiple studies and omics will provide a strong foundation for constructing a validated multibiomarker panel for T2D, an achievement attainable only through collaborative efforts across the scientific community.

## Supplementary Material









## Data Availability

Protein data sets were retrieved from the main text and Supporting Information of the included studies, except for Amorim et al., 2022 (PRIDE: PXD033101) and Wigger et al., 2021 (PRIDE: PXD022561), which were obtained directly from the PRIDE repository. All code used for the different steps of the systematic review and meta-analysis can be found on GitHub, including refinement of the search equation, preprocessing of protein lists, reanalysis of raw data, functional, interaction and semantic analysis, and meta-analysis execution: https://github.com/juliagcurras/metaanalysisT2D. Source code for the provided Shiny apps *pubmedToCsv* and *metaMarkersT2D* is also freely available on GitHub: https://github.com/juliagcurras/pubmedtocsv; https://github.com/juliagcurras/metaMarkersT2D.

## Notes

Supplementary figures and tables mentioned in the Methods and Results sections; Table S1: search equation refinement workflow using the litsearchr R package; Table S2: initial and final search equation after the refinement process; Table S3: inclusion and exclusion criteria followed during the screening; Table S4: QUADOMICs tool adaptation and item requirements; Table S5: detailed description of raw data reanalysis; Table S6: list of relevant biomarkers associated with T2D used to select proteins for the random-effects meta-analysis; Table S7: summary of results for initial and final searches; Table S8: concordances and discrepancies between reviewers in the eligibility assessment of 185 studies; Table S9: detailed preprocessing settings reported for all included studies; Table S10: list of relevant proteins shared by eight or more studies and selected for the random-effects meta-analysis; Tables S11–14: results from a series of post hoc analyses for each protein selected in the random-effects meta-analysis performed to evaluate the robustness and potential biases in the meta-analysis of differential protein expression (S11—outlier detection results, S12—estimates after applying the trim-and-fill method, S13—summary of tests for publication bias, S14—likelihood of publication bias and estimated selection effects results); Table S15: results of subgroup analysis to detect sources of heterogeneity for the three most consistent proteins; Figure S1: Venn diagram showing the overlap of studies retrieved from the three search databases; Figure S2: graphical overview of the included studies based on publication and proteomic characteristics; Figure S3: visualization of demographic and clinical features of study groups (T2D, control) in the final set of studies; Figure S4: number of proteins shared across multiple studies included in the qualitative assessment and the omics-based meta-analysis; Figure S5: results of omics-based meta-analysis computed by type of sample; Figure S6: forest plots illustrating aggregated outcomes from traditional meta-analytical methods for proteins with considerable variability in change direction across studies. (PDF)
Supporting Information 1: results for the different steps of the screening process, databases generated after data extraction, including basal data of the articles and the lists of proteins by article (after processing) (XLSX)
Supporting Information 2: general results of the QUADOMICS tool for each study and item, and extended information on each item (required data, final decision, and additional comments) (XLSX)
Supporting Information 3: detailed information about interaction scores for protein–protein pairs in Figure 3A; results from ORA divided by GO main classes for the 85 proteins shared across more than 7 studies, along with semantic clustering of functional terms; absolute and relative counts computed in the vote counting analysis of proteins shared by more than 6 articles; aggregated FC and adjusted *p*-values resulting from the omics-based meta-analysis for the 2,735 proteins shared by ≥2 studies; contribution and influence analysis for each of the included studies (XLSX)

## Abbreviations

2-DE: two-dimensional electrophoresis
2D-DIGE: two-dimensional difference gel electrophoresis
BMI: body mass index
CI: confident interval
DAA: differential abundant analysis
DDA: data-dependent acquisition
DIA: data-independent acquisition
DN: diabetic nephropathy
DR: diabetic retinopathy
ELISA: enzyme-linked immunosorbent assay
FC: fold change
FPG: fasting plasma glucose
GO: gene ontology
LC: liquid chromatography
log_2_ FC: log_2_-transformed fold change
MALDI: matrix-assisted laser desorption/ionization
MAPE: mean absolute percentage error
MS: mass spectrometry
ORA: overrepresentation analysis
PICO: population, intervention, comparison, outcome
PMC: PubMed Central
SELDI: surface-enhanced laser desorption/ionization
SMD: standardized mean difference
SRM/MRM: selected/multiple reaction monitoring
T2D: type 2 diabetes
WOS: Web of Science
