# Supplementary material for: Exploring Biomarkers in Type 2 Diabetes Mellitus versus Normoglycemia Identified through High-Throughput Proteomics: A Systematic Review and Meta-Analysis
Source: J Proteome Res. 2025 Nov 30;25(1):4–20. doi: 10.1021/acs.jproteome.5c00773 (PMC12772132; doi:10.1021/acs.jproteome.5c00773)
Supplement: Supplementary file 1 [file pr5c00773_si_001.pdf]

Exploring Biomarkers in Type 2 Diabetes Mellitus versus Normoglycemia Identified through High-throughput Proteomics: A Systematic Review and Meta-Analysis

Julia García-Currás[1,2], Raquel Pérez-Lois[3,4], Guillermo L. Taboada[1], María P. Pata[2]*

1. Universidade da Coruña (UDC), Grupo Arquitectura de Computadores, Centro de Investigación en Tecnologías de la Información y las Comunicaciones (CITIC), Elviña, A Coruña 15071, Spain. julia.gcurras@udc.es

2. Biostatech, Advice, Training & Innovation in Biostatistics, Ames (A Coruña) 15895, Spain.

3. Grupo Fisiopatología Endocrina, Área de Endocrinología, Instituto de Investigación Sanitaria de Santiago de Compostela (IDIS), Complexo Hospitalario Universitario de Santiago (SERGAS), Travesía da Choupana s/n, Santiago de Compostela, Galicia 15706, Spain

4. CIBER Fisiopatología de la Obesidad y Nutrición (CIBERobn), Av Monforte de Lemos 3-5, Madrid 28029, Spain.

Table of contents

Additional Supplementary Materials:

- Supplementary material 1 (XLSX). Results for the different steps of the screening process, databases generated after data extraction, including basal data of the articles and the lists of proteins by article (after processing).
- Supplementary material 2 (XLSX). General results of the QUADOMICS tool for each study and item and extended information of each item (required data, final decision and additional comments).
- Supplementary material 3 (XLSX). Detail information about interaction score for protein-protein pairs in Figure S3A; results from ORA divided by GO main classes for the 85 proteins shared across more than 7 studies, along with semantic clustering of functional terms; absolute and relative counts computed in the vote counting analysis of proteins shared by more than 6 articles; aggregated FC and adjusted p-values resulted from the omics-based meta-analysis for the 2,735 proteins shared by >2 studies; contribution and influence analysis for each of the included studies.

Supplementary Tables and Figures in this document:

- Supplementary Table S1. Search equation refinement workflow using the *litsearchr* R package.
- Supplementary Table S2. Initial and final search equation after the refinement process.
- Supplementary Table S3. Inclusion and exclusion criteria followed during the screening.

- Supplementary Table S4. QUADOMICs tool adaptation and item requirements.
- Supplementary Table S5. Detailed description of raw data reanalysis.
- Supplementary Table S6. List of relevant biomarkers associated to T2D used to select proteins for the random-effects meta-analysis.
- Supplementary Table S7. Summary of results for initial and final searches.
- Supplementary Figure S1. Venn diagram showing the overlap of studies retrieved from the three search databases.
- Supplementary Table S8. Concordances and discrepancies between reviewers in the eligibility assessment of 185 studies.
- Supplementary Figure S2. Graphical overview of the included studies based on publication and proteomic characteristics.
- Supplementary Table S9. Detailed preprocessing settings reported for all included studies.
- Supplementary Figure S3. Visualization of demographic and clinical features of study groups (T2D, control) in the final set of studies.
- Supplementary Figure S4. Number of proteins shared across multiple studies included in the qualitative assessment and the omics-based meta-analysis.
- Supplementary Figure S5. Results of omics-based meta-analysis computed by type of sample.

- Supplementary Table S10. List of relevant proteins shared by eight or more studies and selected for the random-effects meta-analysis.
- Supplementary Figure S6. Forest plots illustrating aggregated outcomes from traditional meta-analytical methods for proteins with considerable variability in change direction across studies.
- Supplementary Tables S11-S14. Results from a series of post-hoc analyses for each protein selected in the random-effect meta-analysis performed to evaluate the robustness and potential biases in the meta-analysis of differential protein expression.
    - Supplementary Table S11. Outlier detection results.
    - Supplementary Table S12. Estimates after applying the trim-and-fill method.
    - Supplementary Table S13. Summary of tests for publication bias.
    - Supplementary Table S14. Likelihood of publication bias and estimated selection effects results.
- Supplementary Table S15. Results of subgroup analysis to detect sources of heterogeneity for the three most consistent proteins.

Supplementary Table S1. Search equation refinement workflow using the *litsearchr* R package, including main steps, functions used, selected options, and results obtained.

Steps	Function (*litsearchr*)	Options	Results
**(1)**<br><br>**Find relevant keywords**	*extract_words()*	• A term from title/abstract must appear 5 times at least (from the same document). Method of search: fakerake.<br>• A term from keywords must appear in, at least, 5 documents to be included. Method of search: tagged.	Potentially relevant terms: **654**
**(2)**<br><br>**Build naive network graph**	*create_dfm()*<br>*create_network()*	A term must appear in at least 12 documents (title, abstract or keywords) to be part of the network.	Potentially relevant terms: **221**
**(3)**<br><br>**Filter the coocurrence network**	*find_cutoff()*	Filtered coocurrence network: a cutoff for naive network was established using the cumulative method (retaining 80% of the most relevant nodes), and node importance was measured using the *strength* metric.	Cutoff: **206**<br><br>Potentially relevant terms: **131**
**(4)**<br><br>**Select and group the final terms**	None (manual)	Three groups: outcome (biomarkers); analysis (proteomics) and disease (T2D).	**Relevant terms**: 27
**(5)**<br><br>**Update equation**	*write_search()*	Options: *languages = "English", stemming = TRUE, exactphrase = TRUE.*	Final equation in Supplementary Table S2

Supplementary Table S2. Initial and final search equation after the refinement process.

**Initial search equation**	*("proteomic*" OR "proteomic analysis" OR "LC/MS-MS" OR "label-free SWATH" OR "SWATH-MS" OR "data independent acquisition" OR "DIA" OR "DIA-SWATH" OR "Data dependent acquisition" OR "DDA" OR "shotgun proteomic*")<br>AND<br>("DM2" OR "T2DM" OR "type II diabetes mellitus" OR "diabetes mellitus type 2" OR "type 2 diabetes mellitus" OR "type 2 diabetes" OR "diabetic condition" OR "type II diabetes")<br>AND<br>("biomarker*" OR "significant protein" OR "differential expressed protein" OR "DE protein" OR "protein biomarker")*
**Final search equation**	*("proteomic*" OR "proteomic analysis" OR "LC/MS-MS" OR "label-free SWATH" OR "SWATH-MS" OR "data independent acquisition" OR "DIA" OR "DIA-SWATH" OR "Data dependent acquisition" OR "DDA" OR "shotgun proteomic*" OR "quantitative proteomic" OR "tandem mass spectrometry")<br>AND<br>("DM2" OR "T2DM" OR "type II diabetes mellitus" OR "diabetes mellitus type 2" OR "type* 2 diabetes* mellitus" OR "type* 2 diabetes" OR "diabetic condition" OR "type* II diabetes" OR "type* 2 diabetic patient")<br>AND<br>("biomarker*" OR "significant protein" OR "differential expressed protein" OR "DE protein" OR "protein biomarker" OR "upregul* protein*" OR "downregul* protein*" OR "signific* higher protein*" OR "signific* increased protein*" OR "signific* lower protein*" OR "signific* reduced protein*" OR "express* protein*" OR "protein* express*" OR "protein* level*" OR "protein marker*")*

Supplementary Table S3. Inclusion and exclusion criteria followed during the screening of potential relevant references.

	Inclusion criteria	Exclusion criteria
**1**	High-throughput proteomic analysis of proteome data: DDA (shotgun) and DIA techniques.	Targeted proteomics (SRM, MRM); techniques for quantifying individual protein samples (ELISA, western blot); targeted techniques with quantification based on DNA (SOMAscan) or qPCR (Olink); quantification using gels and staining/fluorescent tags (2D-DiGE).
**2**	Case control, cohort, and observational studies with original data.	Interventional studies.
**3**	Studies with a control group of normoglycemic individuals (adults aged ≥ 18 years).	Diabetes types other than type 2: gestational, type 1.
**4**	Human studies	Non-human studies.
**5**	-	Reviews without original data and book chapters.
**6**	-	Languages other than English.

Supplementary Table S4. Adaptation of the QUADOMICS tool for the current meta-analysis, including the criteria used to answer each item. Possible responses: Yes, No, Unclear.

Item	Description	Requirements
**1. Selection**	Were selection criteria clearly described?	Inclusion/exclusion criteria must have been reported.
**2. Patient spectrum**	Was the spectrum of patients representative of patients who will receive the test in practice?	Lack of sex and age restrictions, and a general source of patients..
**3. Type of sample**	Was the collection procedure fully described?	Required information: Type of sample (serum, plasma, tissue sample, etc.); procedure of tissue sample collection; storage or collection times (time of sample collection, time between sample acquisition and storage); temperature of storage
**4. Physiological and clinical factors**	Were the procedures of biological sample collection with respect to clinical factors described with enough detail?	Mean and standard deviation of age, IBM, diabetes duration, FBG or HBcA1, as well as female/male frequency must have been provided for both T2D and control group. Only missing data in one of the previous variables is allowed to have this item 4 fulfilled.
**5. Pre-analytical procedures**	Were handling and pre-analytical procedures reported in sufficient detail and similar for the whole group?	Detailed description of pre-analytical procedures related with procedure of protein extraction:<br>a) Protein extraction description (defrozen method, vortexed, sonication, centrifugation, quantification);<br>b) Fragmentation (initial quantity, enzyme, buffers and centrifugation cycles)<br>c) Peptide labelling procedure (if it was performed)
**6. Timing to index test**	is the time between the reference standard and the index test short enough to guarantee that the target condition did not change between the two tests?	Intervals between the proteomic test (index test) and the reference standard (diabetes diagnosis) must be short enough (<3 years) to ensure that the patient's condition (T2D) did not change, preserving the validity of the comparison.
**7. Reference standard**	Is the reference standard likely to correctly classify the target condition?	Use of ADA criteria, WHO criteria, HbA1c> 6,5%, fasting blood glucose > 126mg/dL or OGTT 2hPG to determine or confirm the T2D condition.
**8. Verification**	Did the whole sample or a random selection of the sample receive verification using a reference standard of diagnosis?	The reference standard for diabetes diagnosis must have been applied to all patients included in the study.
**10A. Replication Proteomics**	Was the execution of the index test (T2D diagnoses) described in sufficient detail to permit replication of the test (proteomic analysis)?	Required information: Chromatography, Instruments model, Instrument manufacturer, Ionization source, Acquisition mode (for DIA mode, the specific method), Resolution, Mass range, Total time, Voltage information, Fragmentation type, Gas temperature, Scan time, Reference library, FDR for identification, Software.

		Only missing data for 2 items is allowed (out of 15 items for DDA and 16 for DIA) or for 3 items if one of them is Voltage information or Gas temperature (default settings are usually interpreted in these cases).
**10B. Replication of Downstream Analysis**	Was the execution of the index test described in sufficient detail to permit replication of the test?	Required information: software of downstream analysis, Normalization, Statistical test, Multiple test correction method, Imputation method, Functional Analysis. Complete data by article is required to fulfil this item. The following information had to be provided: normalization and imputation, statistical test for differential abundance analysis, p-value adjustment, functional analysis and software.
**11. Execution of reference standard**	Was the execution of the reference standard described in sufficient detail to permit its replication?	Information about procedures and devices used to measure FBG, HbA1c or to perform OGTT test.
**12. Assessment**	Were the index test results interpreted without knowledge of the results of the reference standard?	Discrimination analysis of candidate biomarkers proteins must have been performed and the corresponding classification metrics (AUC, sensitivity, specificity or any equivalent metric), reported.
**16. Validation**	Is it likely that overfitting was avoided? (use of validation cohort or technique)	Validation techniques (Western blot, ELISE, SRM, MRM) must have been used to confirm, at least, one interesting biomarker found by LC-MS/MS.

Supplementary Table S5. Detailed description of the raw data reanalysis performed to generate adjusted p-values and FC for the complete list of proteins compared between T2D and control groups for each study.

Dataset	File location	Re-analysis?	More information
**Vestad et al., 2021**	Supplementary File 2, Sheet *Perseus_All*	Partially	Correspondence between group number and study group was inferred from Figure5C (Group 2 is T2D and Group 1 is control group). Then, potential contaminants were also removed from the database. Finally, log_2FC was estimated, p-values were retrieved from File 2, sheet *Perseus_All* (Student.s.T.test.Significant.2_ column matching the 111 differential expressed proteins reported in the article) and were adjusted using the BH correction.
**Chen et al., 2021**	Supplementary Table S5	Yes	First, proteins with more than 50% of missing data in both groups were filtered out. Then, those with more than 50% missing data in only one group were considered as significant (following the Methods section) and were used in the qualitative analysis with *Amanida*. Then, for the rest of proteins, their quantities were compared between NC (control) and DM (T2D) groups through a t-test, applying a BH correction and log_2FC was also calculated.

**Zou et al., 2020**	Supplementary Excel S1 (tvst-9-13-8_s004.xlsx)	Partially	P-values and FC for Control/T2D provided for all proteins in Excel S1FC. New analysis: FC inversion (to get T2D/Control), log_2FC estimation and adjustment of p-values using the BH correction.
**Amorim et al., 2022**	Txt.zip, proteinGroups.txt (PRIDE PXD033101)	Yes	First, identification of study groups from proteinGroups.txt file was performed. Proteins that were plotted in Supplementary Figure S4 and that showed complete data across the fourth groups were selected. Then, boxplots were displayed and compared with the ones in Figure S4. After that, the correspondence of groups was established: group 2 corresponds with T2D and group 4, with Controls. Second, raw data was preprocessing as follows: 1) reverse database hits were removed; 2) LFQ intensities from T2D (group 2) and Control (group 4) samples were selected; 3) proteins that showed more than 3 missing values in at least one group were removed; 4) LFQ intensities were log_2 transformed; 5) comparisons between the T2D and Control were performed for each protein using a t-test and BH correction, and log_2FC was also obtained.
**Wigger et al., 2021**	Search.txt, sheet proteinGroups.txt (PRIDE PXD022561)	Yes	First, mean of technical replicates (4 by sample) was obtained for each protein, allowing a maximum of 2 missing values. Second, raw data was pre-processed as follows: 1) reverse database, contaminant, and only by site modification identifications were removed from the data set; 2) data was log_2 transformed; 3) only complete data were kept (no missing data allowed); 4) for each protein, T2D and control groups were compared using a t-test with BH correction, and log_2FC estimations were also performed.<br>It is worth to mention that relationship between sample names and groups were inferred using the heatmap in Figure S4e from the original article.
**Skeie et al., 2018**	Supplementary Table S1, Sheet Intensities - DMEC	Yes	In the original article, missing data was neither imputed nor removed, ending with extreme log_2FC values. Therefore, the analysis was repeated and ND group was compared against the rest of the samples (all T2D conditions). No missing data was allowed in any of the previous groups (because ND group only has 4 samples). After this filter, t-test and BH correction were performed, as well as log_2FC estimations.
**Li et al., 2018**	Supplementary Table S1	Yes	Fasting data at baseline was used to compute t-test between DO (diabetic obesity) and NO (normoglycemic obesity) groups, applying BH correction and also performing the log_2FC estimation. Gene name duplicates were removed from the dataset.

Supplementary Table S6. List of relevant biomarkers associated to T2D and explicitly mentioned in the included studies, used to select proteins for the random-effects meta-analysis. Bold text indicates relevant proteins mentioned in more than one study.

Reference	Type Of Sample	Relevant proteins explicitly mentioned on included studies
**Abdulwahab et al., 2019**	Serum	P04004 (VTN); **P02042** (HBD); A8MUN2 (APOB); C9J7Z6 (EIF4E3); A0A087 (recA); P69905 (HBA1); Q08380 (LGALS3BP); Q30KQ8 (DEFB112); D6RA88 (MYL5); **P02766** (TTR); Q9Y4G8 (RAPGEF2); Q96J01 (THOC3); O95342 (ABCB11); O15397 (IPO8); Q5VU13 (SNHG28;VSIG8); O43866 (CD5L); A0A087WXL8 (NA); P05155 (SERPING1); P01859 (IGHG2); H3BTQ6 (TAF1C); Q5TAQ9 (DCAF8); P01861 (IGHG4); Q8N196 (SIX5)
**Amorim et al., 2022**	Eye	**P68871** (HBB)
**An et al., 2018**	Sperm	P29508 (SERPINB3); P07900 (HSP90AA1); P01034 (CST3); P01009 (A1AT;SERPINA1)
**Chen et al., 2020**	Serum	**P00915** (CA1); Q9P2K5 (MYEF2); P01042 (KNG1)
**Kaur et al., 2012**	Serum	P02774 (GC); **P02766** (TTR)
**Lewandowicz et al., 2015**	Urine	P41222 (PTGDS); P98160 (HSPG2); P10451 (SPP1); P60709 (ACTB); P02647 (APOA1)
**Li et al., 2023**	Serum	P08571 (CD14); P26927 (MST1); P27169 (PON1); P00738 (HP); P00748 (F12); B7ZKJ8 (ITIH4); P05362 (ICAM1); P08185 (SERPINA6); P55056 (APOC4); E9PEK4 (CSF1R); Q15485 (FCN2); P62328 (TMSB4X); P62937 (PPIA); Q15582 (TGFBI); P0DP25 (CALM3); P21333 (FLNA); P02749 (APOH); P80108 (GPLD1)
**Nimer et al., 2023**	Serum	**P68871** (HBB); **P02042** (HBD); Q9UNU2 (C4B); P10599 (TXN); P14174 (MIF); P01160 (NPPA); **P00915** (CA1); P00918 (CA2); P32119 (PRDX2); Q06830 (PRDX1); P30041 (PRDX6); P26447 (S100A4); P06703 (S100A6)
**Sachdeva et al., 2024**	Eye	Q9H4F8 (SMOC1); P47972 (NPTX2); Q9Y5W5 (WIF1); P02649 (APOE); Q86VZ4 (LRP11)
**Yan et al., 2024**	Urine	P01011 (SERPINA3); P02768 (ALB)
**Yu et al., 2022**	Plasma	P17936 (IGFBP3)
**Zhao et al., 2021**	Plasma	Q86V24 (ADIPOR2); P0DJI8 (SAA1); P13726 (TF); O95497 (VNN1)
**Zhao et al., 2024**	Liver tissue	P30085 (CMPK1); P06132 (UROD); Q7Z7G0 (ABI3BP); Q8NBP5 (MFSD9); Q9UQ35 (SRRM2); P55084 (HADHB)
**Zou et al., 2020**	Eye	P61626 (LYZ); P25311 (AZGP1); Q13217 (DNAJC3)

Supplementary Table S7. Results for initial and final search using the equations listed at Supplementary Table S2, total articles after combining the results and number of articles after removing duplicates using the DOI as main identifier.

	**Scopus**	**WOS**	**PubMed**	**Total**	**Unique (DOI)**
**Initial search**	470	390	309	969	571
**Final search**	1147	703	572	2422	**1273**

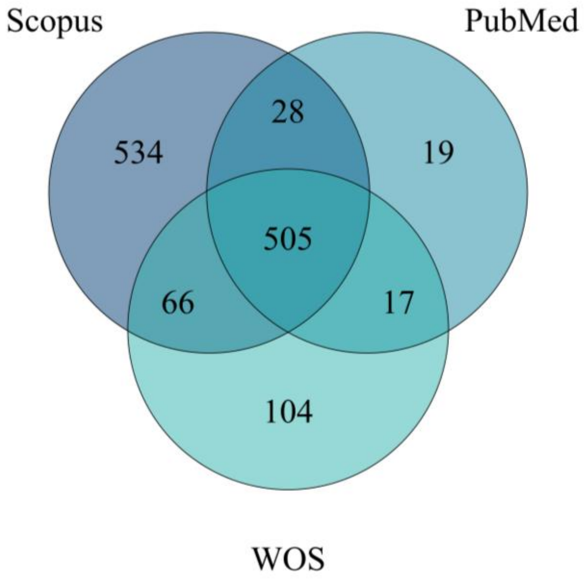

Supplementary Figure S1. Venn diagram showing the overlap of studies retrieved from the three search databases.

Supplementary Table S8. Concordance and discrepancies between reviewers in the eligibility assessment of 185 reports.

		**Decision JGC**		
		**In**	**Out**	**Total**
**Decision RPL**	**In**	32 (17.3%)	6 (3.2%)	38 (20.5%)
	**Out**	13 (7.0%)	134 (72.4%)	147 (79.5%)
	**Total**	45 (24.3%)	140 (75.7%)	185 (100.0%)

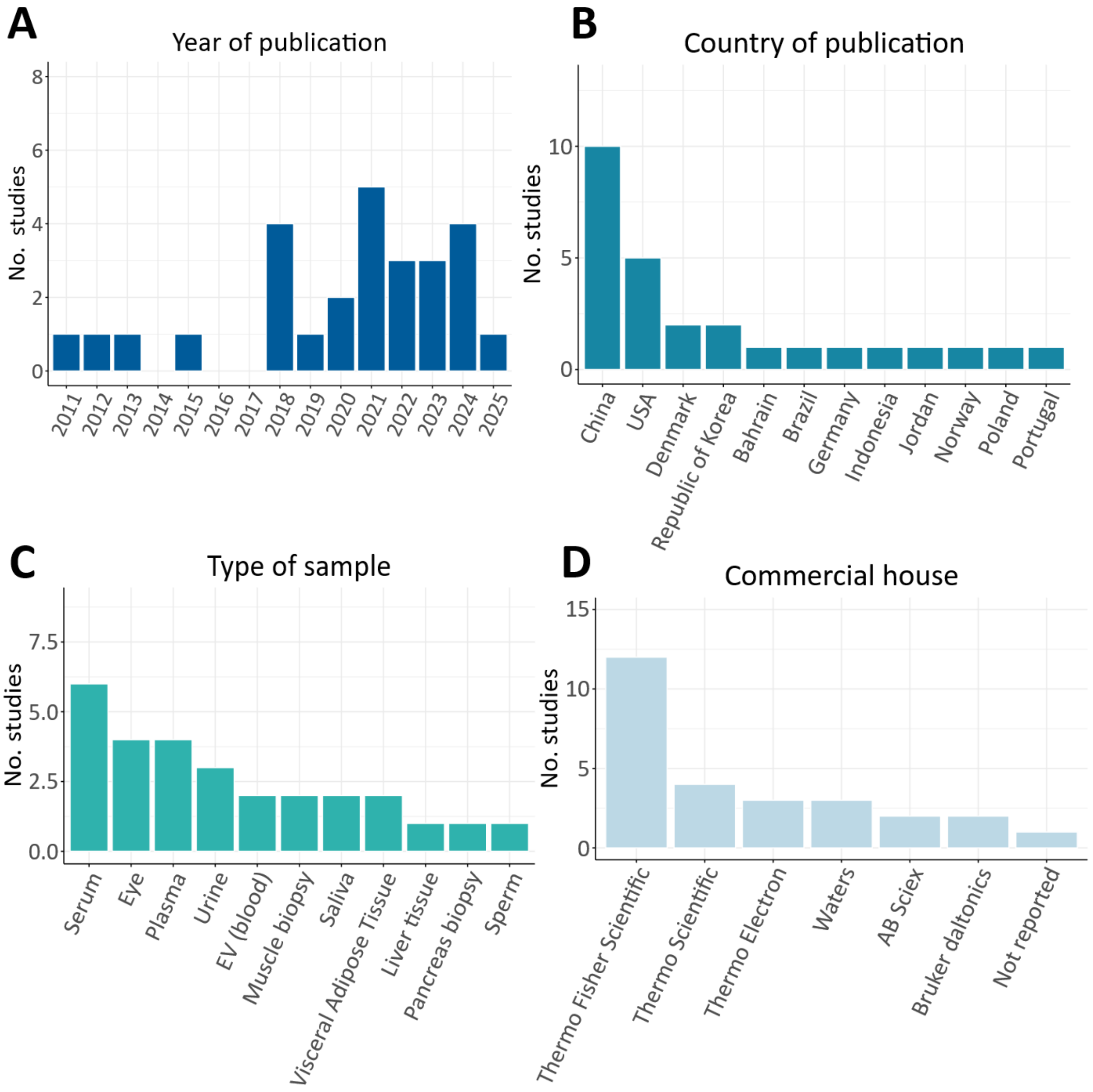

Supplementary Figure S2. Graphical representation of the number of studies by A) publication year; B) country of publication; C) type of sample; D) commercial house, shown as absolute frequencies for the final 27 studies.

Supplementary Table S9. Detailed preprocessing settings reported for all included studies.

Study	Software of analysis	Normalization	Statistical test	P-values adjustment	Imputation
Zhao et al., 2024	R	Not reported	limma	BH	Not reported
Samodova et al., 2025	R;Perseus	Log2	t-test	BH	Yes
Zhao et al., 2021	R	Median	limma	BH	Yes
Carruthers et al., 2021	R	Median	t-test	FDR	Yes
Vestad et al., 2021	Perseus	Log10	t-test	Not reported	Yes
Lewandowicz et al., 2015	MATLAB	Loess	Nonparametric analysis of variance-based resampling test	BH	Yes
Nunez Lopez et al., 2022	R	Log2	limma	BH	Not reported
Chen et al., 2021	R;SPSS	Total intensity	t-test	Not reported	No
Sachdeva et al., 2024	Perseus	Median	t-test	No	Not reported
Zou et al., 2020	SPSS	Not reported	t-test	Not reported	No
Amorim et al., 2022	Perseus	LFQ	t-test	BH	Yes
Thingholm et al., 2011	Not reported	Median	Confident interval	Not reported	Not reported
An et al., 2018	Perseus	Not reported	Not reported	No	Not reported
Nimer et al., 2023	MSstate	Not reported	Linear mixed effect model	Yes (method not reported)	Not reported
Abdulwahab et al., 2019	Progenesis QI V2.0/TransOmics Informatics	Not reported	ANOVA	Not reported	Not reported
Yu et al., 2022	Proteome Discoverer	Not reported	t-test	No	Not reported
Ferreira da Silva et al., 2024	MetaboAnalyst	Not reported	ANOVA	Not reported	Not reported
Chae et al., 2018	Not reported	Quantile	t-test	Yes (method not reported)	No
Kaur et al., 2012	ProteinPilot	Not reported	Not reported	Not reported	Not reported

Chen et al., 2020	R	MinMax	Kruskall-wallis	Not reported	Not reported
Darmayanti et al., 2023	SPSS	Not reported	Confident interval	Not reported	Not reported
Wigger et al., 2021	Perseus	Log	t-test	BH	Yes
Yan et al., 2024	Not reported	Not reported	t-test	BH	Not reported
Li et al., 2023	Proteome Discoverer	Not reported	t-test	BH	Not reported
Skeie et al., 2018	Partek Geonomics	Log10	ANOVA	Not reported	Not reported
Li et al., 2018	Not reported	Not reported	GTLasso	Not reported	Not reported
Kim et al., 2014	Not reported	Quantile	Log median ratio test	Not reported	Not reported

Sample size estimation was not reported in any of the included articles. BH: Benjamin-Hochberg.

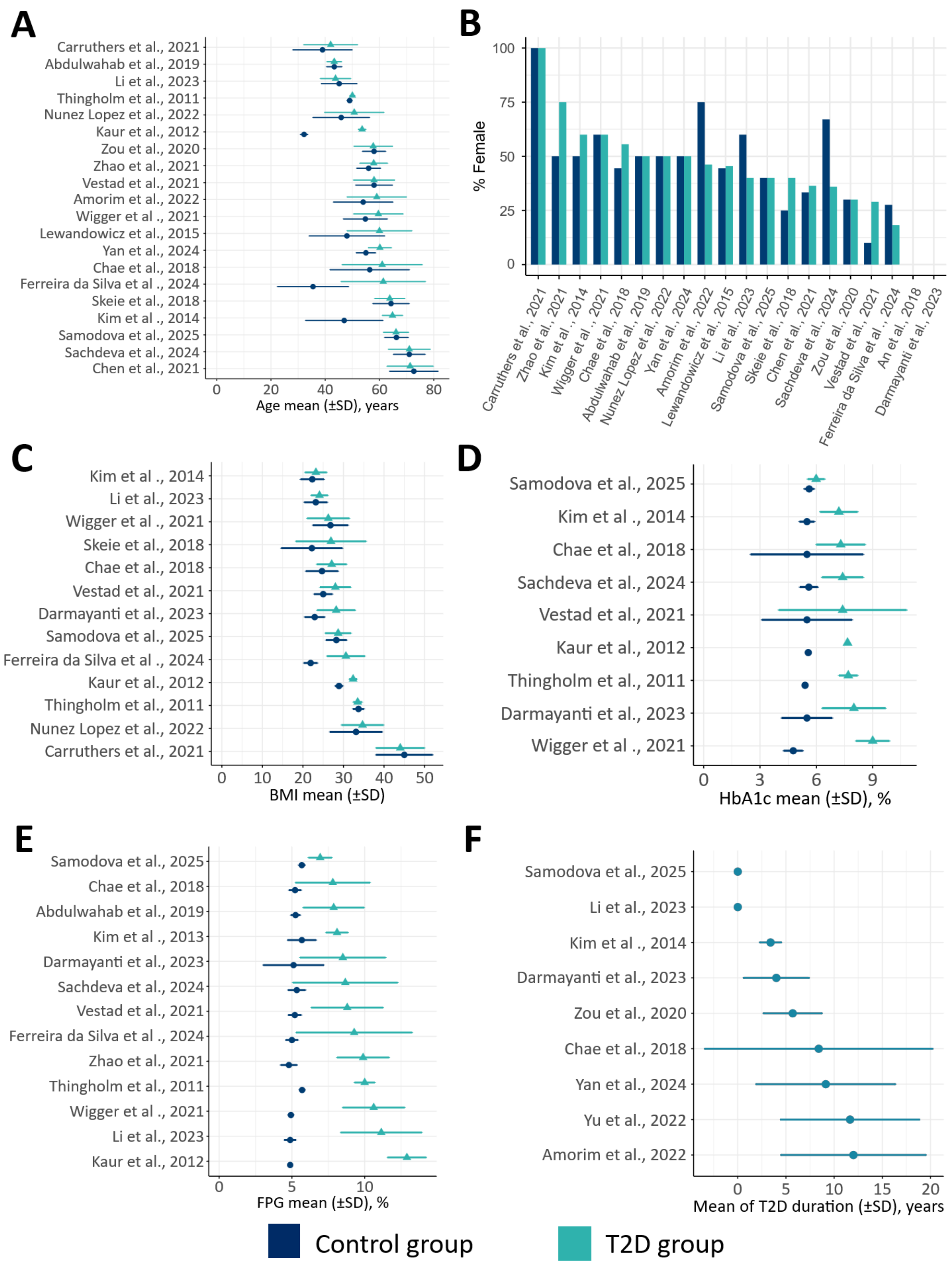

Supplementary Figure S3. Graphical representation of demographic and clinical data by study groups (T2D, control) across the final 27 studies that provided this information. For each study and group, forest plots show the mean and standard deviation for (A) age, (C) BMI, (D) HbA1c,

and (E) FPG. Diabetes duration is similarly depicted for the T2D group only (F). Percentage of women in both T2D and control groups is presented in a bar plot for each study (B).

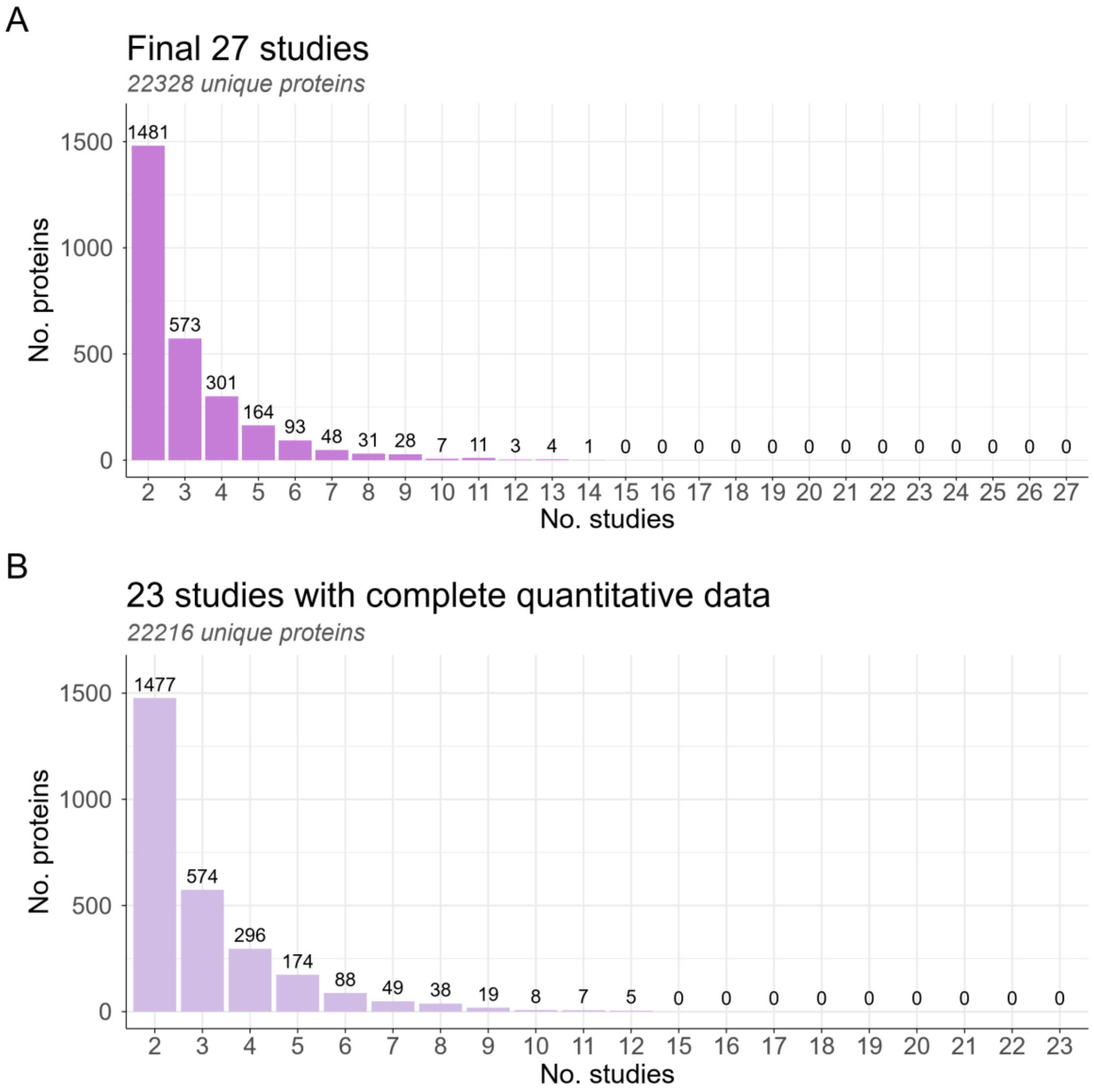

Supplementary Figure S4. Number of proteins shared by more than one article for (A) the final 27 studies included in the qualitative assessment, with 2,745 unique proteins shared by two or more articles; and (B) the 23 studies with complete quantitative data (both p-values and fold changes), used in the omics-based meta-analysis, with 2,735 unique proteins shared by two or more articles.

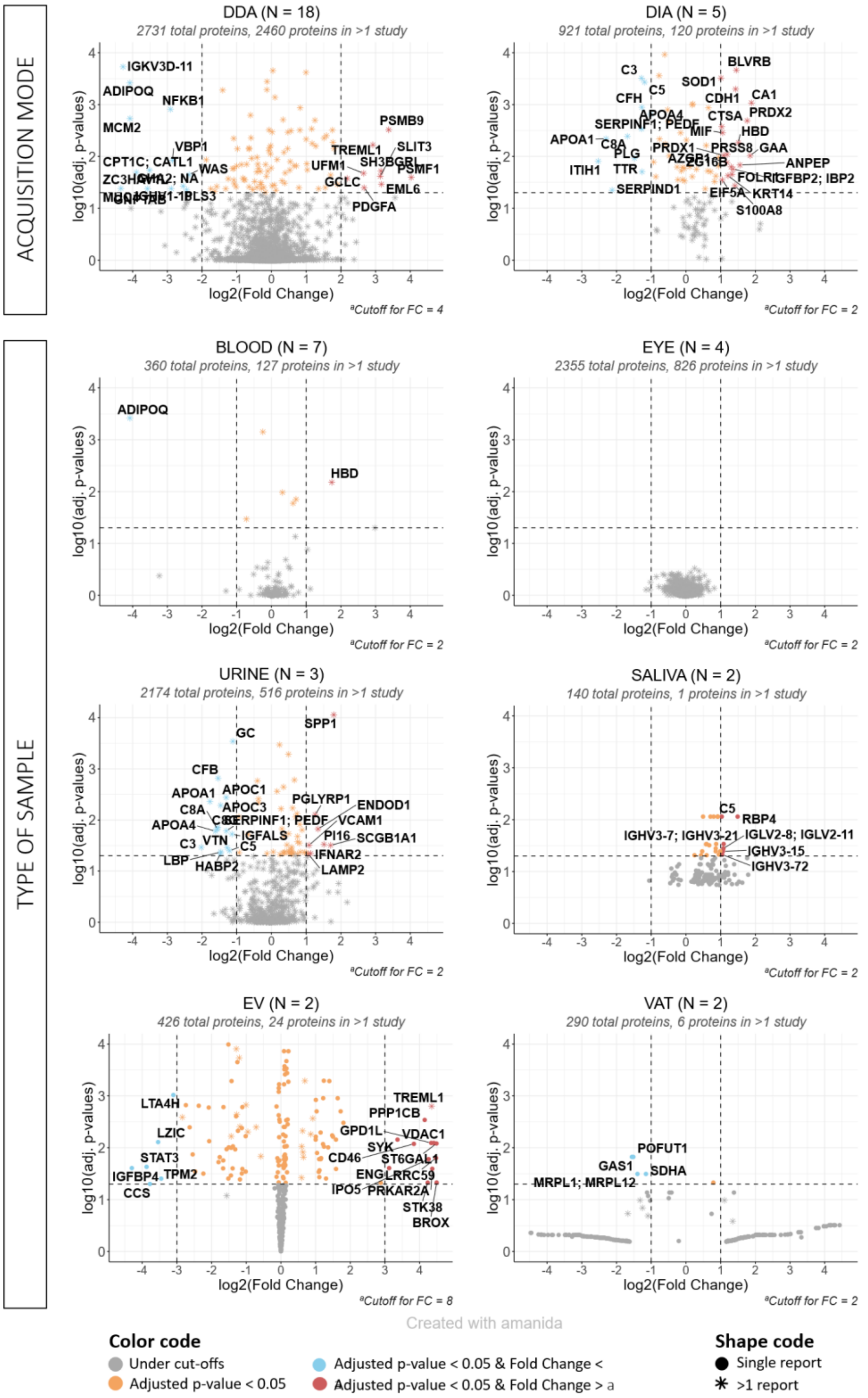

Supplementary Figure S5. Omics-based meta-analysis results grouped by sample type. For blood, eye, and urine, only proteins reported in more than one study were analysed. For the remaining sample types, which include at most two studies each, all proteins were considered.

Supplementary Table S10. List of relevant proteins shared by eight or more studies and explicitly mentioned as potential biomarkers in the literature that were selected for the random-effects meta-analysis.

UniProt ID	Protein name	Gene symbol	Description	(No. studies) References
**P02763**	Alpha-1-acid glycoprotein 1	*ORM1*	Functions as transport protein in the blood stream. Binds various ligands in the interior of its beta-barrel domain. Also binds synthetic drugs and influences their distribution and availability in the body. Appears to function in modulating the activity of the immune system during the acute-phase reaction; Belongs to the calycin superfamily. Lipocalin family.	(10) Li et al., 2018; Yan et al., 2024; Vestad et al., 2021; Chen et al., 2021; Sachdeva et al., 2024; Zou et al., 2020; Amorim et al., 2022; Li et al., 2023; Skeie et al., 2018; Wigger et al., 2021
**P00738**	Haptoglobin alpha chain	*HP*	As a result of hemolysis, hemoglobin is found to accumulate in the kidney and is secreted in the urine. Haptoglobin captures, and combines with free plasma hemoglobin to allow hepatic recycling of heme iron and to prevent kidney damage. Haptoglobin also acts as an antioxidant, has antibacterial activity, and plays a role in modulating many aspects of the acute phase response. Hemoglobin/haptoglobin complexes are rapidly cleared by the macrophage CD163 scavenger receptor expressed on the surface of liver Kupfer cells through an endocytic lysosomal degradation pathway.	(8) Li et al., 2018; Yan et al., 2024; Vestad et al., 2021; Chen et al., 2021; Sachdeva et al., 2024; Zou et al., 2020; Amorim et al., 2022; Li et al., 2023
**P01011**	Alpha-1-antichymotrypsin His-Pro-less	*SERPINA3*	Although its physiological function is unclear, it can inhibit neutrophil cathepsin G and mast cell chymase, both of which can convert angiotensin-1 to the active angiotensin-2.	(9) Li et al., 2018; Yan et al., 2024; Vestad et al., 2021; Chen et al., 2021; Sachdeva et al., 2024; Zou et al., 2020; Amorim et al., 2022; Li et al., 2023; An et al., 2018
**P02647**	Truncated apolipoprotein A-I	*APOA1*	Participates in the reverse transport of cholesterol from tissues to the liver for excretion by promoting cholesterol efflux from tissues and by acting as a cofactor for the lecithin cholesterol acyltransferase (LCAT). As part of the SPAP complex, activates spermatozoa motility.	(9) Li et al., 2018; Yan et al., 2024; Vestad et al., 2021; Chen et al., 2021; Sachdeva et al., 2024; Zou et al., 2020; Li et al., 2023; Skeie et al., 2018; An et al., 2018
**P02649**	Apolipoprotein E	*APOE*	APOE is an apolipoprotein, a protein associating with lipid particles, that mainly functions in lipoprotein-mediated lipid transport between organs via the plasma and interstitial fluids. APOE is a core component of plasma lipoproteins	(8) Li et al., 2018; Vestad et al., 2021; Chen et al., 2021; Sachdeva et al., 2024; Zou et al.,

			and is involved in their production, conversion and clearance. Apoliproteins are amphipathic molecules that interact both with lipids of the lipoprotein particle core and the aqueous environment of the plasma.	2020; Li et al., 2023; Skeie et al., 2018; An et al., 2018
**P04004**	Vitronectin V10 subunit	*VTN*	Vitronectin is a cell adhesion and spreading factor found in serum and tissues. Vitronectin interact with glycosaminoglycans and proteoglycans. Is recognized by certain members of the integrin family and serves as a cell-to-substrate adhesion molecule. Inhibitor of the membrane-damaging effect of the terminal cytolytic complement pathway.	(8) Li et al., 2018; Yan et al., 2024; Vestad et al., 2021; Chen et al., 2021; Sachdeva et al., 2024; Zou et al., 2020; Li et al., 2023; Skeie et al., 2018
**P25311**	Zinc-alpha-2-glycoprotein	*AZGP1*	Stimulates lipid degradation in adipocytes and causes the extensive fat losses associated with some advanced cancers. May bind polyunsaturated fatty acids; Belongs to the MHC class I family.	(8) Li et al., 2018; Yan et al., 2024; Vestad et al., 2021; Chen et al., 2021; Sachdeva et al., 2024; Zou et al., 2020; Amorim et al., 2022; Li et al., 2023
**P32119**	Peroxiredoxin-2	*PRDX2*	Thiol-specific peroxidase that catalyzes the reduction of hydrogen peroxide and organic hydroperoxides to water and alcohols, respectively. Plays a role in cell protection against oxidative stress by detoxifying peroxides and as sensor of hydrogen peroxide-mediated signaling events. Might participate in the signaling cascades of growth factors and tumor necrosis factor-alpha by regulating the intracellular concentrations of H(2)O(2).	(8) Yan et al., 2024; Vestad et al., 2021; Sachdeva et al., 2024; Zou et al., 2020; Amorim et al., 2022; Li et al., 2023; Skeie et al., 2018; An et al., 2018
**P62937**	Peptidyl-prolyl cis-trans isomerase A	*PPIA*	N-terminally processed; PPIases accelerate the folding of proteins. It catalyzes the cis-trans isomerization of proline imidic peptide bonds in oligopeptides.	(8) Yan et al., 2024; Vestad et al., 2021; Chen et al., 2021; Sachdeva et al., 2024; Zou et al., 2020; Amorim et al., 2022; Li et al., 2023; An et al., 2018

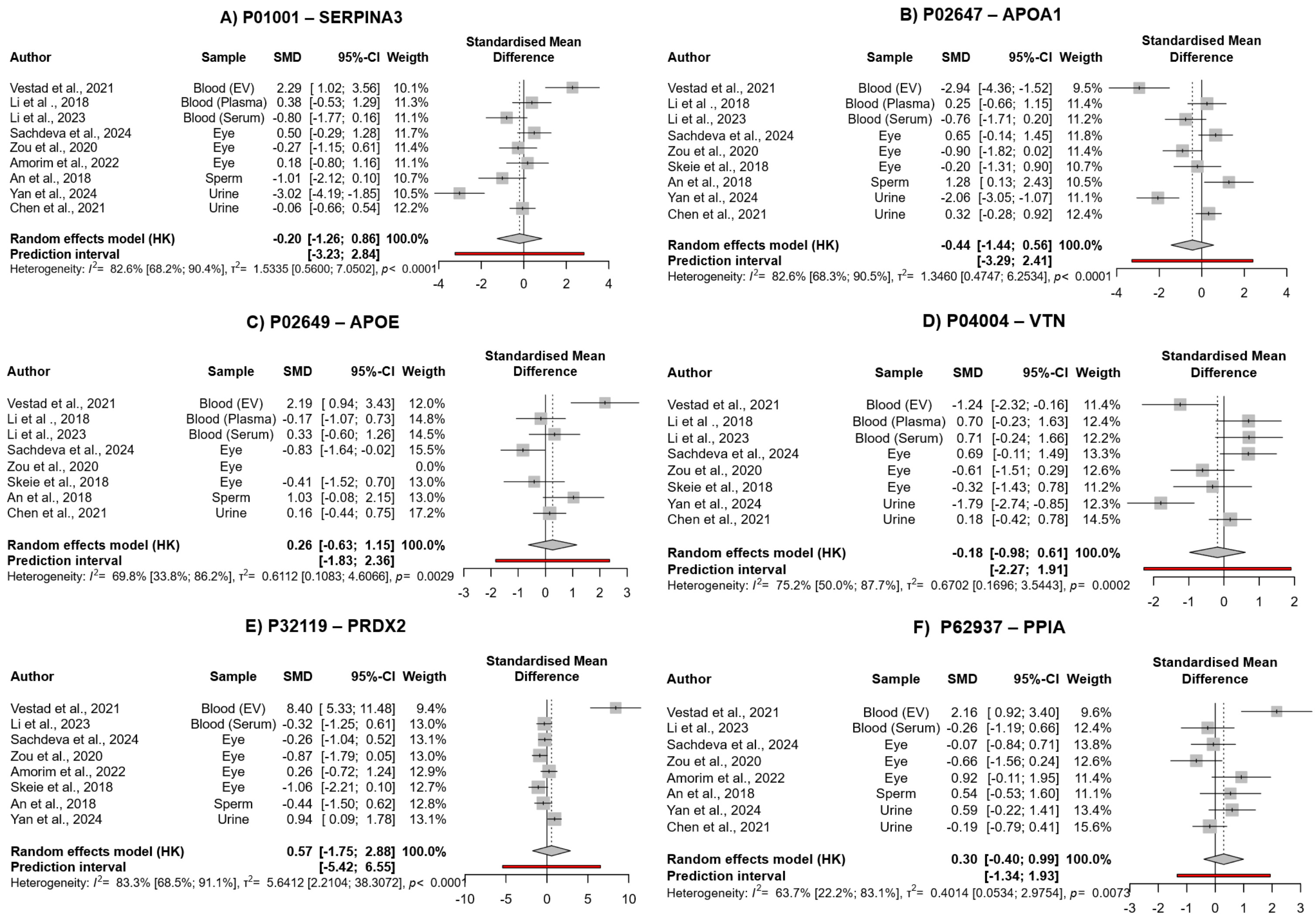

Supplementary Figure S6. Forest plot showing aggregated results from traditional meta-analysis for selected proteins exhibiting high variability in Standardized Mean Difference (SMD) across studies. Individual study effect sizes, 95% confidence intervals, and

corresponding weights are shown alongside the pooled SMD estimated using a random-effects model. Measures of heterogeneity—including I^2 (percentage of total variability due to heterogeneity), τ^2 (between-study variance), and the p-value from the Cochran's Q test—are reported to assess consistency across studies. Positive SMD values indicate higher protein abundance in the T2D group. EV: extracellular vesicles.

Supplementary Table S11. Outlier detection results and changes in heterogeneity statistics (I^2: percentage of total variability due to heterogeneity; τ^2: between-study variance) with and without outliers for each protein showing consistent results across studies. CI: confidence interval.

Protein	Outlier Study	Model	τ^2	95%CI (τ^2)	I^2	95%CI (I^2)
**P02763 (*ORM1*)**	Vestad et al., 2021	With outliers	0.079	0.000–2.076	44%	0%–73.1%
		Without outliers	0.000	0.000–0.247	0%	0%–64.8%
**P00738 (*HP*)**	None	—	0.259	0.000–1.745	56.2%	3.6%–80.1%
**P25311 (*AZGP1*)**	None	—	0.915	0.263–5.266	78.7%	58.3%–89.1%

Supplementary Table S12. Adjusted effect size estimates and prediction intervals from the trim-and-fill method. CI: confidence interval.

Protein	Model	Estimate	95% CI	Prediction Interval
**P02763 (*ORM1*)**	Original	0.448	0.024–0.873	-0.370–1.267
	Without outliers	0.318	-0.095–0.732	-0.133–0.770
**P00738 (*HP*)**	Original	0.469	-0.113–1.051	-0.934–1.872
	After trim-and-fill	0.114	-0.476–0.703	-1.661–1.888
**P25311 (*AZGP1*)**	Original	0.783	-0.126–1.692	-1.756–3.322
	After trim-and-fill	0.352	-0.647–1.351	-2.794–3.498

Supplementary Table S13. Summary of tests for publication bias, including Egger's test, fail-safe N, and the test for excess significance.

Protein	Egger's Test p-value	Fail-Safe N	Excess Significance p-value
**P02763 (*ORM1*)**	0.065	13	0.679
**P00738 (*HP*)**	0.180	7	0.223
**P25311 (*AZGP1*)**	0.086	20	0.048

Supplementary Table S14. Results from selection models assessing the likelihood of publication bias and estimated selection effects. CI: confident interval; LRT: Likelihood Ratio Test.

Protein	LRT p-value	Selection Model Estimate (95% CI)
**P02763 (*ORM1*)**	0.033	6.41 (95% CI: 0.68–60.00)
**P00738 (*HP*)**	0.883	1.68 (95% CI: 0.73–3.86)
**P25311 (*AZGP1*)**	0.565	1.68 (95% CI: 0.56–5.07)

Supplementary Table S15. Results of subgroup analysis to detect sources of heterogeneity for the three most consistent proteins.

Protein	Group	n	SMD	CI95% SMD	p-value	I^2	CI95% I^2	p-value subgroups
**P02763** ORM1	Sample							0.813
	Blood	3	0.310	-2.571-4.823	1.126	0.798	0.361-0.936	
	Urine	2	0.592	-30.135-30.778	0.322	0.293	-	
	Eye	4	0.347	-0.648-1.251	0.302	0.000	0.000-0.847	
	Pancreas	1	0.478	-0.807-1.723	0.458	-	-	
	Data acquisition							0.568
	DDA	9	0.078	-0.066-0.930	0.432	0.485	0.000-0.760	
	DIA	1	0.097	-0.128-1.530	0.701	-	-	
	Quality							0.090
	Low	4	0.918	-3.902-3.967	0.033	0.000	0.000-0.847	
	Moderate	1	0.097	-0.128-1.530	0.701	-	-	
	High	5	0.072	-0.140-1.922	0.891	0.518	0.000-0.823	
	Comercial house							0.875
	Thermo	8	0.104	-0.125-1.030	0.453	0.549	0.002-0.796	
	ABSciex	1	0.097	-0.128-1.530	0.701	-	-	
	Bruker	1	0.478	-0.807-1.723	0.458	-	-	
	Software							0.890
	MaxQuant	5	0.285	-0.804-2.063	0.630	0.711	0.267-0.886	
	Proteome Disc.	3	0.437	-6.077-6.799	0.361	0.000	0.000-0.896	
	Others	1	0.544	-0.766-1.453	0.343	-	-	
	ProteinPilot	1	0.097	-0.128-1.530	0.701	-	-	
**P00738** HP	Sample							0.076
	Blood	3	0.145	-0.799-2.553	0.877	0.291	0.000-0.926	
	Urine	2	0.639	-9.359-8.784	-0.288	0.598	0.000-0.906	
	Eye	3	0.134	-0.616-2.062	0.723	0.000	0.000-0.896	
	Data acquisition							0.004
	DDA	7	0.034	0.072-1.159	0.615	0.206	0-0.64	
	DIA	1	0.076	-1.587-0.080	-0.753	-	-	
	Quality							0.002
	Low	3	0.586	-20.382-20.905	0.262	0.000	0.000-0.896	
	Moderate	1	0.076	-1.587-0.080	-0.753	-	-	
	High	4	0.051	-0.009-1.925	0.958	0.000	0.000-0.847	
	Comercial house							0.004
	Thermo	7	0.034	0.072-1.159	0.615	0.206	0-0.64	
	ABSciex	1	0.076	-1.587-0.080	-0.753	-	-	

	Software							0.015
	MaxQuant	4	0.117	-0.264-1.370	0.553	0.000	0.000-0.847	
	Proteome Disc.	3	0.238	-1.44-3.037	0.798	0.714	0.028-0.916	
	ProteinPilot	1	0.076	-1.587-0.080	-0.753	-	-	
**P25311** AZGP1	Sample							0.700
	Blood	3	0.227	-2.018-4.568	1.275	0.757	0.201-0.926	
	Urine	2	0.619	-11.966-13.271	0.653	0.909	0.676-0.975	
	Eye	3	0.569	-2.477-3.392	0.457	0.811	0.411-0.940	
	Data acquisition							0.125
	DDA	7	0.165	-0.365-1.687	0.661	0.780	0.543-0.894	
	DIA	1	0.001	0.693-2.585	1.639	-	-	
	Quality							0.214
	Low	3	0.492	-1.889-2.740	0.426	0.756	0.193-0.926	
	Moderate	1	0.001	0.693-2.585	1.639	-	-	
	High	4	0.315	-1.457-3.204	0.874	0.825	0.551-0.932	
	Comercial house							0.125
	Thermo	7	0.165	-0.365-1.687	0.661	0.780	0.543-0.894	
	ABSciex	1	0.001	0.693-2.585	1.639	-	-	
	Software							0.173
	MaxQuant	4	0.347	-1.611-3.33	0.86	0.847	0.618-0.939	
	Proteome Disc.	3	0.414	-1.581-2.466	0.443	0.700	0.000-0.912	
	ProteinPilot	1	0.001	0.693-2.585	1.639	-	-	
